# Promoting 21st Century Health and Wellness Skills in Elementary School Children: a Group Randomized Trial

**DOI:** 10.1007/s11121-024-01717-3

**Published:** 2024-08-14

**Authors:** Patrick H. Tolan, Alexis R. Harris, Margaret Burchinal, Patricia A. Jennings

**Affiliations:** University of Virginia, Charlottesville, VA USA

**Keywords:** Social and Emotional Learning, School-based Interventions, Prevention

## Abstract

In a group randomized trial of 45 elementary schools, a whole-child health and wellness curriculum introduced as a regular part of the educational programming was compared to education as usual over 2 years with a 2-year follow-up. The curriculum focused on integrating multiple SEL skills—mindfulness, compassion, and physical awareness—with the intent to advantage developmental patterns for these skills, academic engagement, personal well-being, and student behavior. The program design and trial implementation were launched with end-use delivery and long-term sustainability as integral considerations. Effects were shown for several SEL skills and behavioral indicators previously robustly correlated to long-term outcomes. Effects were demonstrated more broadly in schools serving high-poverty communities, suggesting the impact was stronger where the need was greater. Results are interpreted regarding supporting school-based SEL, the potential of applying group randomized trials with end-use conditions of implementation, and the value of fitting innovation efforts to school system operations, mandates, and priorities for promoting sustainability.

**Supplementary Information:**

The online version contains supplementary material available at 10.1007/s11121-024-01717-3.

The extensive empirical evidence of the efficacy of social and emotional learning (SEL) interventions for child development has set the stage for more attention to implementation features that affect impact and scalability (Domitrovich et al., 2008). Three important considerations are prominent: (1) design attention to implementation features such as staffing, fit-to-schools’ priorities, and existing education budget determinants (Mart et al., 2015); (2) facilitating long-term scalability and sustainability through attention to fit of host systems and aligning of content with influencing priorities of such systems (Fagan et al., 2019); and (3) integrating multiple SEL skill targets into training, including attention to mindfulness and compassion (Jones et al., 2017). This study reports on a group randomized trial that builds from prior research and emergent findings of school-based interventions with intentional consideration of these as interdependent design features.

Reviews and task forces have noted the gap between the potential inferable from efficacy demonstrations and the actual reach and uptake realized for interventions (Durlak et al., 2022; Fagan et al., 2019; Tolan, 2019). For example, Durlak and Dupre (2008) surveyed 600 studies of intervention evaluations and documented substantial variation in effects depending on implementation extent. A Society for Prevention Research task force on scaling up interventions emphasized the importance of system fit, including how funding is secured and distributed, staffing patterns, and policies affecting operations, for implementation adequacy and for sustaining interventions over time (Fagan et al., 2019). A recent review of 12 meta-analyses of SEL programs, while confirming consistent beneficial impact, also found remaining gaps in understanding of and evidence about systematic implementation (Durlak et al., 2022).

Accordingly, each review calls for more consideration of implementation features as part of program tests and emphasizes the value of those that approximate what are approximate conditions for at-scale practice. Intentional integration of these considerations into the content and form of preventive interventions seems important to expedite robust empirical evidence for extrapolation to at-scale and under typical conditions value (Tolan, 2019). For school-based SEL programming, the challenge takes the form of program development that fits existing school resource organization and operational contingencies that are likely to be sustainable within existing system structures and priorities (Cipriano et al., 2023; Mahoney et al., 2021).

The prevailing approach toward viable at-scale programming in prevention science has been to attempt replication of efficacious interventions, with subsequent experiments modifying implementation standards such as who delivers and how controlled the intervention content and processes are (Cook et al., 2019). Through a sequence of step-by-step trial tests for effects under varying conditions of delivery, the extent of fidelity rigor, or for different populations, the goal is yielding knowledge needed for scaling sustainable innovation. However, such an approach can require decades-long programs of research (Glasgow et al., 2012). Small individual-level comparison efficacy studies often do not result in translation to sustainable and effective implementation at scale (Fagan et al., 2019; Tolan, 2019). Shortening the time needed to produce end usable results seems a priority in how evaluation of prevention efforts is cast.

The utility of the accumulated evidence also has been limited by the predominant reliance on trials not using group randomized designs, limiting application for the end-use interest in the impact of whole-school implementation (Cipriano et al., 2023; Pals et al., 2008). Important practical constraints such as budget limitations and difficulty securing enough schools to conduct adequately powered analyses have meant that much of the evidence for SEL programs is from evaluations that were of differences in individuals or between classrooms, not between schools (Pals et al., 2008). The importance of matching evaluation design to implementation is demonstrated by the recent failure of large-scale implementation of efficacious programs to produce significant benefits with programs previously showing effects with individual or classroom-level comparisons (e.g., Troncoso & Humphrey, 2021). Improving the applicability of evidence for to-scale implementation may rest in part on applying group randomized designs for valid inference about population impact (Khine, 2022; Sussman et al., 2006).

The importance of such considerations for improving the usability and applicability of intervention findings can be seen in research on school-based programming incorporating mindfulness practices to promote youth development. Over the past two decades, there has been growing interest in school-based mindfulness programs and research on the impacts of such programs (Roeser et al., 2020). A meta-analysis of 66 randomized controlled trials of mindfulness-based programs (MBP) delivered to children in school and clinical settings (*N* = 20,138; *n* = 9552 MBP, *n* = 10,586 controls) (Dunning et al., 2019) found that MBP resulted in small but significant improvement compared to controls in attention, mindfulness, executive functioning, negative behavior, depression, and anxiety/stress. However, many of the studies utilized small samples, many were demonstration efforts not integrated into the school operations, and few could assess effects via multi-level modeling that can account for school systems’ hierarchical structure (e.g., students nested within classrooms, nested within schools), recommended as best practice in educational research (Khine, 2022). In contrast, a large cluster randomized controlled trial (43 control, 42 intervention schools) in secondary schools in the UK, testing an adaptation of mindfulness-based cognitive therapy (MBCT; Segal et al., 2002), did not yield results consistent with smaller efficacy-focused trials. Over the study, rigorous implementation quality and fidelity monitoring were used, and effort was made to ensure integration into school operations. In comparison to the standard social and emotional learning programming (practice as usual), no significant benefits were found (Kuyken et al., 2022). Moreover, among students at risk of mental health problems, the SBMP condition participants scored more poorly on some outcomes (Montero-Marin et al., 2022). This contrast of findings from this large-scale implementation group randomized trial and the effects from individual-level demonstration trials brings into relief the value of integrating appropriate experimental rigor under conditions approximating at-scale implementation and sustainability.

School-based program design can now benefit from the accomplishments of the SEL movement, primarily via the organization of the Collaborative for Social and Emotional Learning (CASEL), in linking constructs from diverse interventions into a coherent framework (CASEL, 2013). In addition, recent systematic reviews have shown a relation between multiple competencies and impact (Cipriano et al., 2023). CASEL has rendered a model of five distinct but interrelated core competencies that have strong empirical support: Self-Management, self-awareness, social awareness, relationship skills, and responsible decision-making (Lawson et al., 2019). Empirical support for the framework has been demonstrated, albeit with some variation in how constructs are labeled and operationalized (McKown et al., 2009; Ross & Tolan, 2018). Jones et al. (2017) independently reached a similar conclusion from a conceptual review of SEL, elaborating to include skills embodied in mindfulness practices and compassion practice programs. These advances were the framework for CSP program content and approach. The Compassionate Schools Project promotes multiple SEL skills through a lens of child health and wellness, integrating a focus on compassion, mindfulness, and movement practices.

## The Compassionate Schools Project

The Compassionate Schools Project took advantage of the accumulated efficacy demonstrations and lessons learned about advantageous implementation to design a SEL-focused health and wellness curriculum to be implemented in schools as a class which is part of the regular education schedule. With the intentional integration of system-friendly implementation and sustaining features, we tested effects using a longitudinal growth analysis of a group randomized design. The Compassionate Schools Project (CSP) curriculum *Flourish* (Harris & Jennings, 2023) integrates SEL with physical activity, health promotion, contemplative practices developmentally tailored for children (including somatic, sensory, and movement-based practices), and compassion promotion (Mahoney et al., 2021). The intervention was designed for elementary school (Grades K–5) to be implemented universally as a part of health and physical education. Implementing as a regular for-credit course already mandated was seen as facilitating integration into school operations (e.g., priorities, scheduling, staffing, facility use) and being congruent with institutional regularities such as educational standard mandates. Teaching the course required training (curriculum content and implementation guidance) provided as professional development that counted toward teachers’ annual professional learning requirements. Moreover, the curriculum was designed by education experts, consulting state and national guidance for health education and SEL (see supplemental materials for the detailed description of the curriculum and standards relied on). CSP teachers’ employment was by school administrators following the personnel policies of the school system, with consultation from our curriculum experts.

## The Present Study

The present report focuses on intent-to-treat analyses of a 5-year group randomized controlled trial of 43 elementary public schools in a system located in a mid-sized US city that serves an economically and ethnically diverse population. We hypothesized that CSP condition students would show improved growth trajectories over the intervention and follow-up period (4 years of assessment) compared to those in the education as usual condition in SEL skills, subjective well-being and peer skills, behavior and adjustment, academic competence and achievement, and discipline referrals. We were also interested in whether there may be differential impacts for subgroups of students. We hypothesized that students in schools with greater challenges (measured by student poverty level) would have more benefits. We also examined if benefits for outcomes differed by gender or grade level at first exposure to the curriculum.

## Method

This longitudinal study began in 2016 and included all children in participating elementary schools (Grades K to 5). Schools and a sample of students were followed up to 4 years from baseline with an intended design of two waves of data collection (fall and spring) each school year (8 waves). The sample was comprised of two cohorts of schools that started in successive years. This report focuses on student outcomes from direct assessments, student self-reports, and teacher ratings of the randomly selected student sample.

### Study Sample

The sample for this trial was 43 K–5 elementary schools. Within these schools, 4762 students participated. Participating schools were randomized to the CSP condition or to the education as usual (EAU) comparison condition. Table 1 lists school characteristics by condition, and Table 2 lists student distribution across grades at baseline.

**Table 1:** School characteristics and CSP intervention

	EAU					CSP					Comparison	
	*N*	Mean	Std	Min	Max	*N*	Mean	Std	Min	Max	SMD	*p*
*Total enrollment*	20	472.5	113.0	239.0	732.0	23	464.7	102.4	275.0	703.0	.07	.81
*Percentage of student body*												
*Free and reduced-price lunch*	20	67.58	16.32	26.20	89.10	23	74.18	13.73	32.70	93.80	.44	.16
*Limited English proficiency*	20	14.17	13.20	0.18	41.32	22	15.50	10.75	0.18	40.44	.11	.72
*White*	20	38.33	18.52	11.82	73.65	23	38.14	17.04	2.70	72.02	.01	.97
*Black*	20	36.45	18.22	9.74	71.93	23	38.60	20.32	9.29	88.89	.11	.72
*Hispanic*	20	15.29	9.56	2.39	28.72	23	13.41	6.37	1.37	25.74	.23	.45
*Other*	20	9.93	3.25	3.85	14.98	23	9.84	4.44	2.40	23.11	.02	.95
*Reported school data*^*a*^												
*Proportion suspended*	20	0.07	0.10	0.00	0.40	23	0.08	0.09	0.01	0.35	.11	.70
*Math mechanics*^*b*^	20	40.12	15.20	8.10	70.00	23	39.76	12.17	6.70	61.10	.03	.93
*Reading mechanics*^*b*^	20	41.74	14.45	10.10	73.30	23	41.07	12.37	14.70	69.10	.05	.87

*EAU* Education as Usual School, *CSP* Compassionate School Project School, *SMD* standardized mean difference

^a^From year prior to enrollment in study

^b^Percentage proficient or better

**Table 2:** CSP intervention group and initial grade

	Grade						
	K	1	2	3	4	5	Total
	*N* (%)	*N* (%)	*N* (%)	*N* (%)	*N* (%)	*N* (%)	*N* (%)
*EAU*	367 (7.8%)	371 (7.9%)	342 (7.3%)	360 (7.7%)	369 (7.9%)	335 (7.1%)	2144 (45.6%)
*CSP*	441 (9.4%)	431 (9.2%)	424 (9.0%)	420 (8.9%)	418 (8.9%)	421 (9%)	2555 (54.4%)
*Total*	808 (17.2%)	802 (17.1%)	766 (16.3%)	780 (16.6%)	787 (16.8%)	756 (16.1%)	4699 (100%)

*EAU* Education as Usual Comparison Group, *CSP* Compassionate Schools Project Group

#### School Selection

School recruitment was completed in two consecutive school years, with the goal of recruiting up to 50 elementary schools from a total population of 97 elementary schools. District leadership determined that schools that were currently under a state improvement plan at the time of recruitment were not eligible. Recruitment communications explained that the intervention schools would receive training and support for the new curriculum and funding for an additional teacher to implement the curriculum. EAU-conditioned schools would receive an annual budgetary incentive to apply for the improvement of their offerings related to SEL, health and wellness, and the first opportunity for implementation post-trial. School principals agreed (1) to enter a lottery for school randomization, (2) to maintain a Health and PE teacher throughout the study period, (3) to provide space and time for the implementation of the new curriculum if an intervention school, and (4) to hire a qualified teacher to implement the curriculum if selected as an intervention school.

All eligible elementary schools were stratified using cluster analysis into subgroups with comparable characteristics based on the proportion of white students and of students with free and reduced lunch. These two factors were used for blocking because other characteristics did not provide further significant differentiation. Randomization resulted in 23 interventions and 20 EAU conditions; see the CONSORT Diagram (Fig. A in the Supplementary Information) and details of randomization in the supplementary material.

#### Teacher and Student Selection

Within each of the 43 participating schools, two classrooms at each grade level were randomly selected for participation in student data collection. Those classroom teachers received a consent packet for each student along with small incentive items (pencils) for every student who returned a signed form (whether or not the parent/caregiver agreed to their participation). Upon returning 80% of their consent forms, participating classrooms received a classroom incentive (snacks). Classroom returned consent rates ranged from 5 to 100%, with a mean of 69%. Of the returned consents, the classroom proportion of parents who consented to have their child enroll in the study ranged from 20 to 100%, with a mean of 80%. Research staff randomly selected 5 boys and 5 girls (when possible) from each of the 2 participating classrooms (20 children per grade level) to participate in the study.

### Data Collection Procedures

Study protocols were approved by the Institutional Review Boards of the university and the partnering school district. The study design was collecting 8 waves of data over 4 years, in the fall and spring of each school year. In the first year, the process was delayed, so Cohort 1 baseline data was collected in the winter. Pandemic-related school closures resulted in the inability to complete the remaining waves, ending data collection at Wave 8 for Cohort 1 and Wave 6 for Cohort 2.

#### Child Assessments

All participating students in one grade level were assessed at the same time over two sessions. For student self-reports, means were computed for scales when at least 80% of items were completed.

#### Teacher Surveys

Teacher surveys were obtained through an online survey platform or by paper surveys delivered to their schools. Teachers received $5 per completed survey. For teacher reports, means were computed for scales when at least 80% of items were completed.

#### School Records

School archives were accessed through the school district office.

### Intervention Procedures

The intervention consisted of the school-wide implementation of the health skills curriculum, *Flourish* (Harris & Jennings, 2023), training for implementing teachers, and additional implementation support. The curriculum was implemented in each school across each of the whole school years with the expectation of about 80–100 min per week of core lesson instruction, usually in 2 lessons per week as the Practical Living (PL; health and physical education) class. Each school was staffed by one PL teacher who had formerly taught health and physical education and one PL teacher who was hired specifically because of this project.

#### The Flourish Curriculum

The CSP Flourish Curriculum is a health and wellness curriculum for children in Grades K–5 (ages 5–11) that integrates SEL, mindful awareness practices, movement, and skills and mindsets for healthy eating (Harris & Jennings, 2023). The curriculum has three elementary grade bands (K–1, 2–3, 4–5) organized into seven units. Like other empirically tested SEL-centered programs, Flourish focuses on cultivating competencies such as self-awareness, Self-Management, social awareness, relationship skills, and responsible decision-making (CASEL, 2013), with activities promoting mindsets of mindfulness, compassion, and body awareness integrated throughout. All lessons included interactive SEL activities, “mindful moments” (e.g., calming breaths, focusing exercise, gratitude/appreciation) practices, mindful movement, and rest and reflection time. Some lessons included activities to encourage healthy eating, drawing from research on child nutrition and eating competence (Satter, 2007).

In the first year of each 2-year curriculum, teachers were coached to provide the lessons as completely as possible, focusing on the core activities/learning objectives. In the second year of each band, the lessons are repeated with differentiation opportunities and extensions for repeated practice and deeper learning. Schools were provided with all materials needed for curriculum implementation. A detailed description of the curriculum is in the supplemental material.

#### Training and Implementation Supports

Training, consultation, and coaching were provided for the duration of the study. Training for implementing teachers included a 5-day (30-h) professional development institute prior to the implementation and a 1-day mid-year booster. Coaches supported implementing teachers through weekly visits and monthly professional learning community meetings. PL teachers in the EAU-conditioned schools received professional learning and coaching support as typically provided by the district. CSP schools were also offered, based on voluntary participation. The Community Approach to Learning Mindfully (CALM for Educators) program is open to all its faculty. CALM is an evidence-based program of brief (20 min) sessions before the start of the school day that includes practice with mindfulness, breathing, intention-setting, and movement (Harris et al., 2016). Details of training and other implementation supports for each condition are provided in the supplemental materials.

## Measures

We outline measures utilized in this study below, including score scaling. Full description of the measures, scoring details, and psychometric characteristics are provided in the supplement.

### School and Child Demographic Characteristics

#### School Level Demographic Characteristics

Rates of free and reduced-priced meals, racial and ethnic composition, proportion of English-language learners, state math and literacy test scores, and rates of suspensions were obtained from school-level archival district data. For moderation analyses, we split schools into groups of those with 80% or more free or reduced lunch rates (Digest of Education Statistics, 2019).

#### Child Level Demographic Characteristics

Student gender, age, and racial/ethnic group (categories defined by the district) were obtained from parents or from school records. We split grade at first exposure into *early* (in kindergarten, 1st or 2nd grade at outset) and *later* (3rd, 4th, or 5th at outset). Gender was binary (male or female).

### SEL Skills

SELWeb (McKown et al., 2016) direct performance measures, student self-report, and teacher ratings of students were utilized. SELWeb was administered to Grades K–3, but Grades 4 and 5 only received the Emotion Recognition and self-control modules. A related online direct assessment, VESIP (Virtual Environment for Social Information Processing; Russo-Ponsaran et al., 2018), was used for age-appropriate assessment of social problem solving in Grades 4–8.

#### Emotion Recognition

Responses on 40 SELWeb Emotion Recognition (Grades K–5) items were assigned a score of 2 (correctly labeled), 1 (neutral label), and 0 (incorrect label), and an overall score was computed as the mean of the items.

#### Self-Management

SELWeb *Frustration Tolerance* (Grades K–5) assessed the number of correct (max = 23) object-matching trials completed during a 90-s frustration challenge, awarding 1 point for each correct response. SELWeb *Choice Delay* (Grades K–5) was a ten-trial test where higher scores were awarded for more valuable self-control-based choices, up to a maximum of 30 total points.

#### Attention and Inhibitory Control

The Flanker test (Grades K–8; Diamond et al., 2007) of attention and inhibitory control was scored based on average response time (RT) in milliseconds (ms) for accurate responses on the mixed trial (most challenging) and across all trials (overall score).

#### Social Problem-Solving

SELWeb Social Problem Solving (Grades K–3) assessed participants’ social reasoning in response to questions based on illustrated vignettes. VESIP social problem-solving (Grades 4–8) assessed similar social reasoning dimensions in response to animated social situation vignettes. For both SELWeb and VESIP, composite social problem-solving scores were computed as mean scores across all vignettes.

#### Social Self-Efficacy

*VESIP Social Self-Efficacy* (Grades 4–8) was measured as the average self-rated confidence score (1–5 Likert scaling) across 5 social problem-solving scenarios.

#### Empathic Concern

Empathic concern (Grades 4–8) was the average score on the 7-item empathic concern subscale of the Interpersonal Reactivity Index (IRI; Davis, 1983) adapted for use with elementary-age children (Catherine & Schonert-Reichl, 2011).

#### Teacher Rating of Student SEL Skills

Classroom teachers (not the PL teachers) rated students in Grades K–5 using the Teacher Report on Students’ Social and Emotional Competence (CASEL & the American Institutes for Research, 2013). An overall score was computed as the mean rating across 20 items. Scaling basis is described in detail in the supplement.

### Subjective Well-Being and Sense of Peer Community

#### Subjective Well-Being

Subjective well-being (Grades 3–8) was assessed using the mean score of the 7-item *Students’ Life Satisfaction Scale* (SLSS, Huebner, 1991).

#### Sense of Peer Community

Sense of peer community (Grades 4–8) was assessed using the mean score on a 5-item measure of sense of community in school (Battistich et al., 1997), adapted to measure the sense of community among peers, specifically (Madill et al., 2014).

### Behavior and Adjustment in School

#### Behavior

Students’ behavior and adjustment in school (Grades K–5) were rated by classroom teachers (not PL teachers), utilizing the Strengths and Difficulties Questionnaire (SDQ; Goodman, 1997). We recorded subscale scores of the average rating for each student for *Emotional Symptoms* (5 items); *Conduct Problems* (5 items), and *Prosocial Behavior* (5 items)*.*

#### Academic Competence

Academic competence (Grades K–5) was rated by classroom teachers using the Academic Competence Evaluation Scale-Short Form (ACES-TS; Anthony & DiPerna, 2018). We recorded a mean of four subscales (motivation, engagement, study skills, and interpersonal skills) to render an overall score for the ACES, referred to as *Academic Enablers*.

### School-Level Academic Achievement and Discipline Referrals

For each participating elementary school, we recorded the percentage of students who scored at the proficient level or above in reading and math skills each year and the suspension count per year recorded in school records. These counts were transformed into rates (total suspension count/total enrollment).

### Implementation Integrity

CSP classes were observed to monitor implementation integrity. The proportion of 9 items indicating whether specific lesson elements were implemented during the observation class period was recorded. Across all teachers and observations, the average score was 0.73 (median = 0.78), indicating substantial adherence to the curriculum.

## Results

### Preliminary Analyses

Consent rates for schools were compared for demographic differences, the proportion of students with free and reduced lunch, and by grade. None was significantly different by condition, except for a slightly higher consent rate for schools with a higher proportion of Hispanic children in CSP (*r* = 0.25) than in the EAU group (*r* =  − 0.06). Full analyses are in the supplement. Condition comparisons of demographic characteristics of the schools and students at baseline did not differ significantly, except that there were more students from high-poverty schools in the EAU condition (33% compared to 24% CSP condition; see Table C in supplemental materials). Because there was a variation between conditions in school in free or reduced-price lunch and Hispanic student proportion and because each was more than 15% of a standard deviation, these both were included in models as covariates (What Works Clearinghouse, 2018).

Differential attrition was tested, comparing baseline scores among those retained and not at wave 6. The rationale and method of comparison are detailed in the supplemental material. Small in magnitude but significant differences were found in the baseline Self-Efficacy score (higher for retained in CSP condition, lower for retained in EAU) and the Empathic Concern score (higher for retained in CSP, no significant difference for retained vs. lost in the EAU condition). No other baseline scores differed by retention at wave 6 (Table D of supplemental material).

### Outcome Analyses

More extensive explanation of the specific analytic models is provided in the supplemental material. Essentially, inferential analyses were based on 3-level longitudinal hierarchical linear models (HLM) of growth curve slopes of the linear rate of change over time from baseline for student outcomes. Nesting of children in schools, at baseline, was accounted for by estimating school-level random intercepts and slopes from the individual coefficients. Time was centered at the end of year 2 (i.e., time 4), so the main effect parameters estimated growth centered at the end of the intervention period. The unconditional fixed effect model included the cohort and the child’s intervention condition (of school at baseline), with linear and quadratic change terms over time. Conditional model added terms for (1) the proportion of the school that received a free- or reduced-price lunch (FRPL), (2) the proportion of white, and (3) the proportion of Hispanic as covariates and interaction terms for three hypothesized categorical moderators: (1) poverty status of school at random assignment (high poverty category if 80% or more of students FRPL rate), early vs later grade of a student at the trial outset (K–2 or 3–5), and gender. The three moderators were crossed with the intervention condition, time, and time squared. We dropped non-linear growth and non-linear moderation interactions from final models if not significant in initial runs. Multiple imputation was used for estimating gender (17% missing). Analyses based on teacher ratings included a clustering variable to account for shared classroom among some students. For school-level archive comparisons, 2-level HLM analyses were utilized with growth for each school (level 1) with intervention condition and cohort (level 2).

### HLM Analyses of Student-Level Outcomes

#### Social Emotional Learning Skills (SEL)

**Fig. 1:**
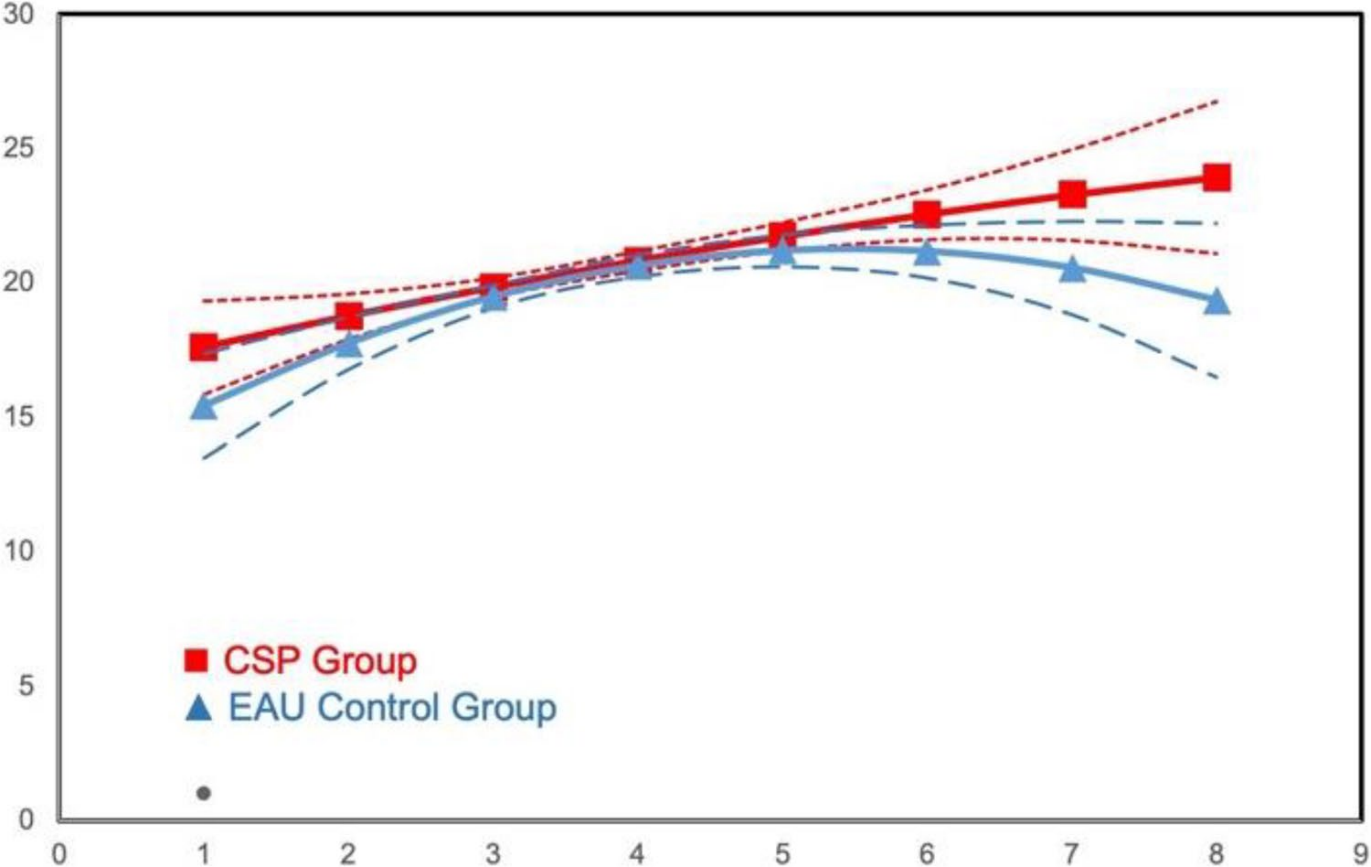
Intervention effect on Delayed Choice moderated by grade: means by wave for students who entered the study in Grades K–2

**Fig. 2:**
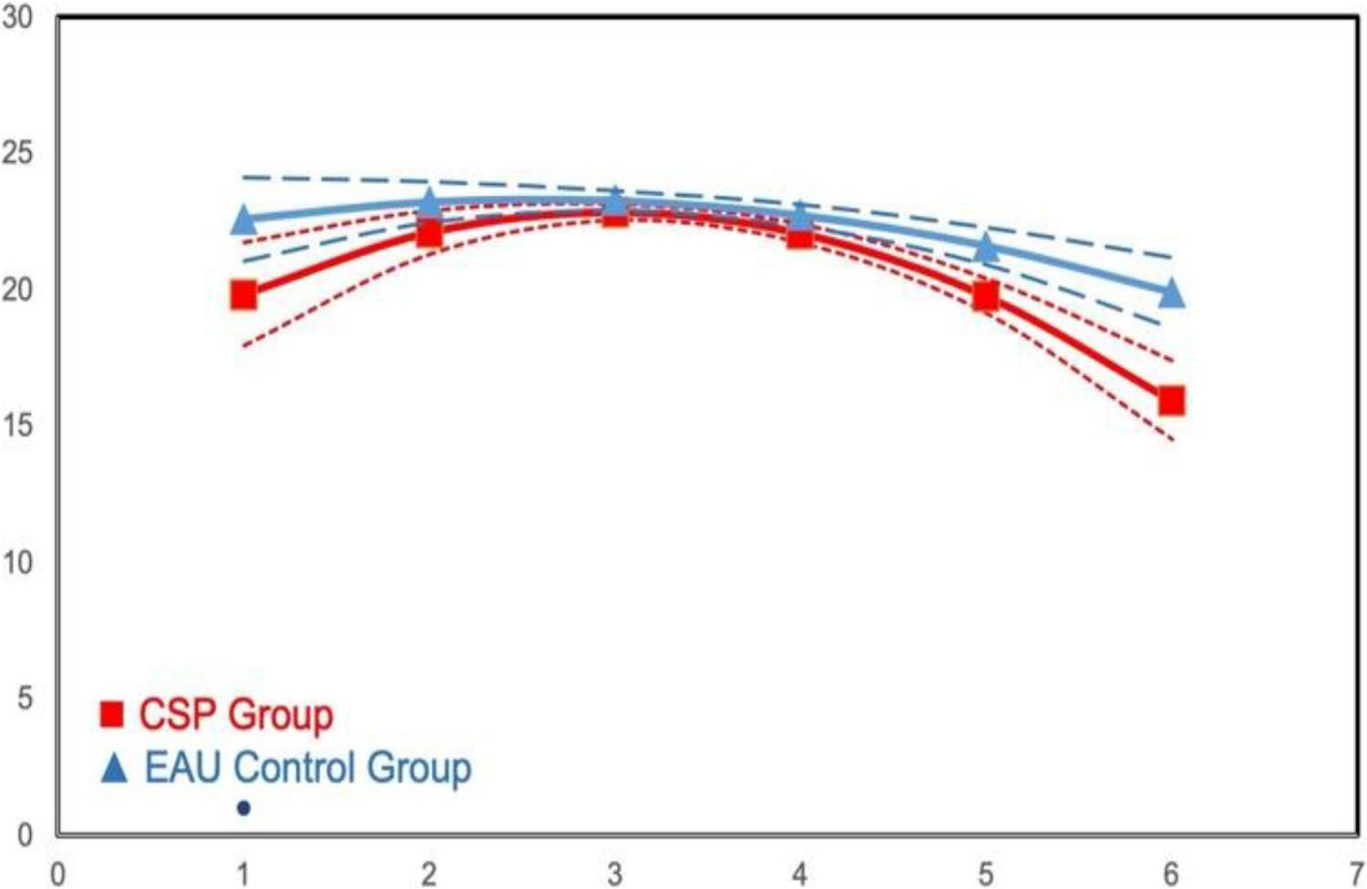
Intervention effect on Delayed Choice moderated by grade: means by wave for students who entered the study in Grades 3–5

Table 3 lists results for Emotion Recognition and Self-Management skills. ITT growth model analyses revealed no significant difference for Emotion Recognition. Among the tests of Self-Management, comparison for *Choice Delay* main effects was not significant, but moderation analyses indicated CSP × initial grade × time interactions in linear growth (*B* = 0.83, *se* = 0.28, *p* < 0.01). CSP students showed improved performance over time compared to EAU students if they were older (Grades 3–5) when the project began, but not if in earlier grades at the outset. Among students first exposed to the CSP class at later grades, a limited decrease over time is observed in the EAU condition, while CSP students grow in differentiation toward lower scores over time (see Figs. 1 and 2, respectively). No significant effects were found for Frustration Tolerance.

**Table 3:** HLM results: SEL skills; Emotion Recognition and Self-Management

	Emotion Recognition		SW Choice Delay	SW Frustration Tolerance	Flanker Overall Response Time		Flanker Mixed Response Time	
	*B* (se)	*B* (se)	*B* (se)	*B* (se)	*B* (se)	*B* (se)	*B* (se)	*B* (se)
Intercept	0.356*** (0.027)	21.824*** (0.178)	21.714*** (0.165)	18.16*** (0.068)	18.046*** (0.052)	796.814*** (3.299)	891.346*** (4.811)	890.184*** (3.816)
Cohort	0.242*** (0.037)	− 0.327 (0.241)	− 0.31 (0.195)	− 0.456*** (0.085)	− 0.3*** (0.068)	− 4.505 (4.538)	− 8.146 (6.549)	− 4.46 (5.169)
Time	0.308*** (0.03)	0.042 (0.14)	0.01 (0.137)	0.306*** (0.079)	0.447*** (0.084)	− 40.846*** (1.534)	− 5.529*** (1.329)	− 5.434*** (1.003)
Time^2^	0.039*** (0.008)	0.064 (0.056)	− 0.353*** (0.065)	− 0.495*** (0.021)	− 0.515*** (0.024)	6.716*** (0.629)	− 1.158*** (0.242)	− 1.033*** (0.211)
CSP	− 0.053 (0.037)	− 0.407 (0.238)	− 0.304 (0.224)	− 0.043 (0.091)	0.008 (0.07)	− 5.055 (4.536)	− 9.387 (6.462)	− 5.652 (5.167)
CSP × time	− 0.001 (0.04)	− 0.214 (0.188)	− 0.301 (0.18)	− 0.089 (0.107)	− 0.095 (0.114)	− 6.39** (2.076)	− 5.018** (1.775)	− 4.511*** (1.347)
Grade (K-G2 = 1)			− 2.082*** (0.218)		− 0.93*** (0.072)	55.465*** (3.479)		− 11.649** (4.199)
Grade × time			1.741*** (0.217)		0.604*** (0.069)	− 8.673*** (2.193)		8.897*** (1.473)
Grade × time^2^			0.718*** (0.143)		− 0.223*** (0.05)	− 5.465*** (1.227)		
CSP × grade			0.828** (0.28)		0.007 (0.09)	2.866 (4.445)		− 3.005 (5.669)
CSP × grade × time			0.707** (0.272)		0.016 (0.088)	− 5.324 (2.967)		− 1.886 (1.996)
High pov School			− 0.022 (0.042)		0.041 (0.133)	0.437 (8.821)		− 2.208 (10.058)
Pov school × time			0.044 (0.037)		0.149 (0.191)	9.915** (3.606)		7.072** (2.307)
CSP × pov school		− 0.062 (0.063)	− 0.082 (0.5)	− 0.11 (0.155)		8.801 (10.296)		6.213 (11.725)
CSP × PovSch × time		− 0.024 (0.09)	− 0.406 (0.396)	− 0.175 (0.25)		− 7.512 (4.684)		− 5.815 (2.997)
CSP × PovSch × time^2^								
Gender (male = 1)		− 0.13*** (0.03)	− 0.255 (0.202)	− 0.184** (0.065)		− 23.414*** (3.315)		− 25.659*** (4.29)
Gender × time		− 0.044 (0.025)	− 0.348 (0.183)	0.040 (0.057)		1.465 (2.46)		− 2.964 (1.532)
CSP × gender		− 0.047 (0.04)	0.149 (0.273)	− 0.048 (0.085)		2.477 (4.505)		− 6.177 (5.844)
CSP × gender × time		− 0.011 (0.034)	0.084 (0.252)	− 0.092 (0.077)		− 4.206 (3.716)		− 1.064 (2.079)
CSP × gender × time^2^								
School % FRPL		− 0.002 (0.001)	− 0.024* (0.009)	− 0.007* (0.003)		− 0.052 (0.222)		− 0.197 (0.25)
School % White		0.002 (0.001)	0.01 (0.008)	0.004 (0.003)		0.206 (0.191)		0.62** (0.215)
School % Hispanic		0.001 (0.002)	− 0.012 (0.013)	0.005 (0.005)		0.287 (0.317)		0.352 (0.357)

^*^p < .05; **p < .01; ***p < .001

Model 1 included cohort, CSP, time, and time-squared and crossed CSP with time and time-squared. Nonsignificant interactions involving time-squared were dropped

Model 2 added gender, initial grade, and high-poverty schools and interacted them with CSP, time, and time-squared. Nonsignificant interactions involving time-squared were dropped

**Fig. 3:**
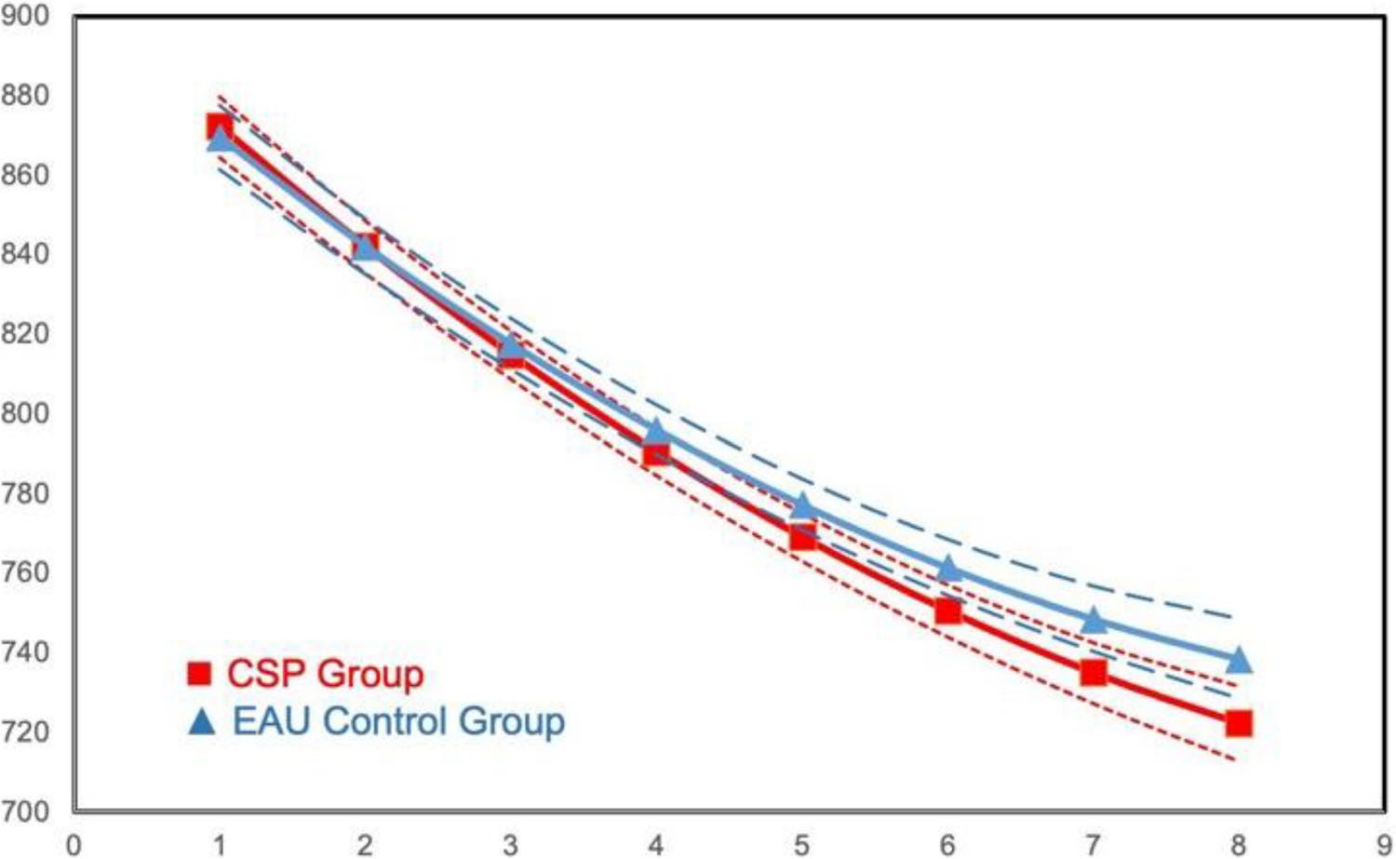
Intervention main effect on Flanker average overall response time across trials: means by wave

**Fig. 4:**
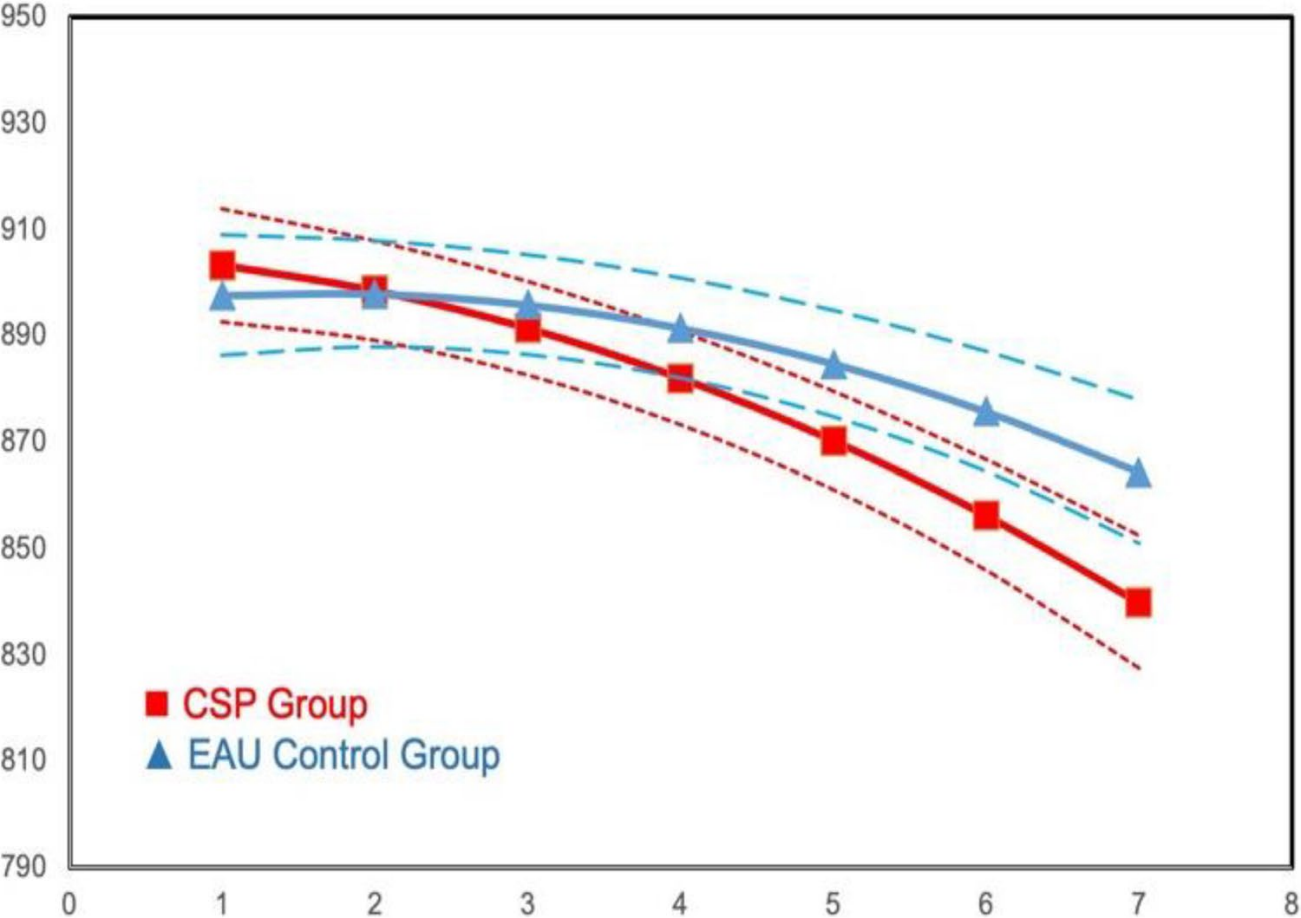
Intervention main effect on Flanker average response time for the Mixed Trial: means by wave

ITT growth model analyses of the Flanker test of attention control yielded significant main effect group differences for both Overall Trials (*B* =  − 5.44, *se* = 2.34, *p* < 0.05) and Mixed Trials (*B* =  − 5.02, *se* = 1.78, *p* < 0.0; see Table 3). The CSP students showed greater decline over time than the EAU students, reflecting greater improvement in attention over time. Model results and illustrative growth figures for condition differences are presented in Figs. 3 and 4, respectively.

This table and these figures provide details of the SEL skills analysis. Due to space limitations, the tables of results and growth figures for subsequent analyses are in the supplement. We describe here all results and interpretation of significant differences.

Analyses of SELWeb *Social Problem Solving* indicated no intervention-related differences in Grades K to 3. For the *Social Information Composite* on the VESIP assessment (administered in Grades 4–8), there was not a significant main effect, but moderation analyses showed differential CSP impact by school poverty level (*B* = 0.04, *se* = 0.02, *p* < 0.05). Among high-poverty schools, the CSP-conditioned students showed continuous gains in this skill, while the EAU-conditioned counterparts skill level remained essentially unchanged. No significant difference was identified among low-poverty schools (see Supplemental Table H and Figs. B and C).

No significant differences were found for Teacher-rated SEL skills.

#### Self-Efficacy and Empathic Concern

ITT growth model results for Self-Efficacy indicated a significant main effect of more accelerated growth over time for the CSP students (*B* = 0.06, *se* = 0.01, *p* < 0.01). CSP condition students showed a continuous increase over the measurement period while the EAU counterparts’ level tapered off. Moderation tests were not significant (Supplemental Table H and Figs. D and E). No significant differences were found for *Empathic Concern* (Supplemental Table I).

#### Student Subjective Well-Being and Sense of Peer Community

The CSP and control groups do not differ in *Subjective Well-being*. Ratings of *Sense of Peer Community* differed by condition in form but not linear slope (*B* =  − 0.04, *se* = 0.02, *p* < 0.05). CSP students started with lower ratings with little change over time, but the EAU group showed decline until the last year (*B* =  − 0.04, *se* = 0.02, *p* < 0.05); see Table I and Fig. D).

#### Student Behavior and Adjustment in School

ITT analyses by condition of teacher ratings of student behavior revealed no significant main effects. However, moderation models revealed differential effects depending on school poverty level for Conduct Problems (*B* =  − 0.04, *se* = 0.02,* p* < 0.01) and Prosocial Behavior (*B* = 0.08, *se* = 0.03, *p* < 0.05). Among students in high-poverty schools, teachers rated CSP students as showing lower levels of Conduct Problems and higher levels of Prosocial Behavior over time, while levels of ratings of EAU students Conduct Problems and decreased Prosocial Behavior over time (Supplemental Table J, Figs. F, G, H, and I).

Grade at first exposure moderated condition differences in slope change over time in Prosocial Skills, with interactions between initial grade, CSP condition, and time (*B* =  − 0.07, *se* = 0.03, *p* < 0.05) and between initial grade, CSP condition, and time-squared (*B* =  − 0.05, *se* = 0.02, *p* < 0.05). These interactions indicate different patterns of growth and the overall extent of growth over time by condition for younger and older students. Among students who entered the study in Grades 3–5 (older), CSP students show increasing scores from baseline, while EAU condition counterparts show decreasing levels. The opposite pattern is found for the younger (K–2) students. Prosocial Skills also was the single instance of moderation of treatment differences by gender (*B* = 0.03, *se* = 0.01, *p* < 0.05). At the end of treatment, the difference between the CSP and EAU students was slightly larger for males than for females (Supplemental Table J, Figs. J, K, L, and M).

No differences by condition occurred for ACES scores (Supplemental Table J).

#### School Achievement Proficiency and Suspension Rates

No differences were significant by condition for school records rates of academic achievement proficiency (math and reading) and suspensions (Supplemental Table K).

## Discussion

This study provides results pertinent to three important prevention issues. First, the group randomized ITT analyses add to the support previously shown for the value of SEL and compassion/mindfulness-focused school-based interventions for facilitating healthy development and reducing problem behaviors and other important risk markers (Cipriano et al., 2023; Jones et al., 2017). This curriculum integrates the five skills identified in the CASEL framework (2013) with the additional emphasis on closely aligned mindfulness and compassion strategies. As suggested by Jones et al. (2017) and others, programs that attend to multiple SEL skills are likely to be more effective and preferable for promulgation (Cipriano et al., 2023; Durlak et al., 2022). As CALM was offered on a voluntary basis for teachers in the CSP condition, that may have contributed to the benefits.

In this case, applying an elementary school SEL-focused health and wellness course curriculum, we found positive benefits in developmental patterns for attention control, Self-Efficacy, and perceived peer support, with additional effects for those students in high-poverty schools for social problem-solving, prosocial behavior, and behavior problems. For some outcomes, the effect was to enhance the rate or extent of development, and in others, it was to maintain capabilities, preventing what would be otherwise deleterious developmental processes. As has been the case in other SEL intervention trials, we found effects for some targeted skills and not for others. This is consistent with the observation by Cipriano et al. (2023) in their review of the past 40 years of SEL programs that no program has reported effects on all assessed skills. In this case, three measures showed significant main effects, with more revealed with moderation by school poverty conditions. Further examination, including why some related skill measures showed effects when others did not and how implementation variations affected impact would aid in understanding value fully.

Lending credence to the positive findings is that many of the SEL effects here come from direct performance assessments using a measure derived to represent the CASEL framework, and other effects were based on ratings from teachers not involved in the delivery of the program, with ratings by differing teachers over time. Moreover, effects were significant for skills found to be among the most salient in reviews tracing predictors of long-term behavioral, academic, and social outcomes such as attention/self-control (Moffitt et al., 2011), Prosocial Behavior (Jones et al., 2017), and conduct problems (Patterson et al., 1989).

School poverty level was a moderator for several outcomes, suggesting the impact was broader and greater in schools serving populations with greater need, lagging in resources, and typically evidencing poorer student academic and social outcomes. School income inequality is perhaps the most substantial differentiating determinant of learning and social outcomes (National Academies of Science, Engineering, and Medicine and National Academies Press, 2023; Reardon et al., 2019). That the intervention can show more benefits in these communities where the need is greater bolsters the potential value of this intervention. The benefits for students in high-poverty schools included problem-solving, conduct problems, and prosocial behavior. Levels of problem behavior are a prominent concern in school management and in high-poverty schools in particular. Moreover, the results rendered here are maintaining positive developmental capabilities, not simply curtailing problems. This program seems to promote resilience capabilities.

We found one instance of moderation of effects by age of students at first exposure. Most notably, the social problem-solving advantage was evident for CSP students first exposed in Grades 3–5. These effects align with when the development of the skills is most rapid and individual differences are most likely to be evidenced (McKown et al., 2009). However, as different measures were applicable for earlier grades than later, the difference may reflect measurement variation. It may also be that the curriculum fit to the needs of different grades for this skill varied, although test of that is beyond the capability of these data.

Inherent in testing intervention is that the implementation characteristics cannot be differentiated from content, nor can one distinguish what aspects of the content or delivery methods are responsible for differences. However, this unavoidable limitation was viewed as showing the importance of intentionally integrating implementation and curriculum form and substance. For example, any interpretation of our results includes recognizing the CALM program as part of the CSP condition. It has been empirically associated with benefits for educator mindfulness, well-being, and Self-Efficacy. In this case, CALM involvement was voluntary and generally limited, and participation varied widely among CSP schools. However, we cannot determine to what extent effects reflect its inclusion. Another inseparable aspect of this evaluation study was the provision of support for one of the two CSP teachers in each intervention school. This presents a potential impact due to the difference in personnel involved in the Practical Living class by condition. At present, the benefits are attributable to the Flourish Curriculum and implemented with ancillary accompaniments and the staffing level difference. However, we note that this is an inherent limitation of experimental trials and is an important basis for intentional attention to implementation that approximates end-use conditions and facilitates sustainability.

The second area of prevention research this study was intended to inform is the translational interest in the efficiency of advancement from efficacy and effectiveness demonstrations to scalability. Most specifically, the approach here was spurred by the extensive time required when the approach is to work in steps from initial conception to large-scale demonstrations for readiness for scaling. When the need is so urgent and extensive, the decades of time needed to progress to knowledge for utility is a serious impediment. At this point, there is ample empirical demonstration of the value of SEL school-based programs, and there are many programs from which next-stage muti-component programming can be fashioned (Durlak et al., 2022; van de Sande et al., 2019). As we reasoned in our conceptualization and implementation, the extensive efficacy demonstrations and lessons learned through systematic reviews, and the accumulating experience about challenges of implementing viable and sustainable efforts in schools provide a justification for engaging more immediately in larger-scale community field trials undertaken with conditions like those of actual end. This study provides one example of how expeditious end-use applicable results can be achieved and the value gained from GRCTs toward these goals.

Another consideration spurring this effort is that the translation of findings from well-controlled and often small-scaled experimental trials has been confounded by the accompanying differences in operational control and delivery systems characterizing the implementation. By conducting trials with end-use operational and implementation methods and systems in a GRCT, increased confidence can be accorded about applicability. For school-based interventions, GRCTs have an advantage given the likely scale-level implementation and the differences found in at least one recent large GCRT (Kuyken et al., 2022). The positive findings here elaborate on that contrast, suggesting that it is not just a matter of analytic design or control of the implementation. However, there are other differences that may account for our positive findings in contrast to Kuyken et al. (2022) such as this being a program designed specifically for elementary school students and the test by Kuyken et al. (2022) being an adaptation of an adult program and CSP being a part of the regular curriculum and that intervention being an add-on/special program.

Because of the extensive work over the past four decades, the identification of important issues in the implementation and their relation to impact its heightened attention is needed to implementation that fits with critical considerations in what schools prioritize, engage with readily, and can sustain (Durlak et al., 2022; van de Sande et al., 2019). This study represents such an approach. The elaboration in implementation attention applied here, and perhaps one applicable for prevention efforts more broadly, was to integrate end-use conditions and their relation and constraints on implementation into the design of the program at the outset of the randomized controlled trial. By design, the staffing and scheduling requirements were set up to fit the existing school periods and use of space, with staffing by certified teachers employed by the school system and working within the career tracks and union seniority systems. These choices were intended to not require extraneous responsibilities for personnel or specialized management approaches. The intent was that the Flourish Curriculum serve as a standard class within the ordinary school day as part of regular education to become an integral part of the school’s functioning and priorities.

The third area of intended pertinence for this study was the usability and sustainability of tested school-based programming for social-emotional learning and healthy development. As noted by multiple reviewers and commissions, this has been a vexing impediment for prevention in general (Cipriano et al., 2023; Fagan et al., 2019). While the roster of programs showing potential benefits has grown, the uptake into actual practice and school system ownership and financing has lagged greatly (Durlak et al., 2022). Many reasons have been identified in reviews, commission reports, and commentaries (Tolan, 2019). We highlight three that informed the present case that seemed to have aided in the continuation and expansion of the programming after the initial funding and demonstration period. First is informing the program delivery from the system priorities and contingencies that can determine what the school could prioritize and maintain, including relying on internal providers, the role of professional career distinctions (e.g., teachers following career tracks), responsibility distributions (e.g., specific subjects, not extra time or different duties), and administrative structures (e.g., building-level decision making and subject supervisory lines, union and district agreements). Similarly, attention was given to the importance of governmental mandates for educational priorities and the use of time and school space for what was likely to be sustained. The goal was to fit into how schools must operate.

Second, the funding for the delivery and management of the programming while initially partially external (one of two teachers funded by the demonstration effort) was set to phase out from the outset, with an agreement for absorption into the school budgeting process after the 2-year implementation. As intended the schools came to view, the teachers as part of their faculty, the course as a set part of their curriculum, and the funding as part of their budget. When we finished our funding for the evaluation study, most schools chose to keep the position and funding became part of the general district budget. Moreover, in subsequent years, the school system has expanded the program and funding to other schools.

The feature of this trial of funding an additional teacher may not be necessary for implementation, however. The staffing needs would depend on school size. Also, schools could offer the course only in certain grades, decreasing the overall staffing need. Some trial schools sustaining the program have done so with one teacher for the school, decreasing the exposure per year. Other districts have implemented the program without hiring new teachers.

Third, this approach is an alternative to a whole school approach and the accompanying requirements of engagement that the approach has typically entailed (Goldberg et al., 2019). While the whole-school approach has many attractive qualities and a recent meta-analysis has documented the benefits of that approach, that review acknowledged the substantial requirements of buy-in and staffing rearrangement that are inherent in a whole-school approach (Durlak et al., 2022; Goldberg et al., 2019). By engaging the school through offering a curriculum that can be implemented as a health and physical education that is a part of the regular education already, less dependence on broad consensus and wholesale shift of priorities were necessary to implement. In the present approach, the value of the programming seems demonstrated by the observed broadening of acceptance over the 2 years of implementation and beyond, and the sustaining and expansion post the formal trial. While we observed the spread of the values and practices in many schools over time, we did not have to attain such buy-in as a prerequisite. The support and felt ownership grew and spread as others observed what they considered a positive impact.

## Limitations

Several limitations should be noted. Among the most important was that the CSP condition included implementation features and ancillary efforts such that any inference of impact carries with it an assumption of application with these features. This inherent limitation of any trial is a primary impetus for the intentional inclusion of such features with an orientation to end-use/at-scale procedures. In addition, there were some differences at baseline by condition, albeit small in extent and controlled for in the analyses. The design used carried limitations on how extensively students were followed over the potential assessment period, which although tempered in impact by the school-level randomization, still merits consideration for confidence accorded the results. Also, only 3 tests yielded significant results, with the most results showing for high-poverty schools. While effects were found across areas, this pattern argues for due caution in confidence accorded the results. While the effects spread across major areas of intended impact, the inconsistency of findings in some areas needs also to be recognized.

## Conclusion

This GRCT was intended to test a school-based universal intervention to support whole-child development in the form of a SEL-focused health and wellness curriculum for elementary school children that integrated mindfulness, compassion, and movement. Utilizing accumulated empirical knowledge and experience-based lessons from earlier interventions the purpose was to test for efficacy of an approach that also incorporated setting priorities and operational contingencies to promote integration and sustainability. These initial ITT analyses suggest some important benefits for outcomes linked to risk and long-term outcome in prior studies and with impact more evident for students in high-poverty schools. These attributable effects are limited to the implementation approach used here, including voluntary access to CALM for teachers in the CSP schools. The approach also has implications for how implementation, scalability, and sustainability might be considered in designing and testing future prevention efforts.

## Supplementary Information

Below is the link to the electronic supplementary material.

Supplementary file1 (DOCX 117 KB)
