# Supplementary material for: Promoting 21st Century Health and Wellness Skills in Elementary School Children: a Group Randomized Trial
Source: Prev Sci. 2024 Aug 14;25(6):919–33. doi: 10.1007/s11121-024-01717-3 (PMC11390807; doi:10.1007/s11121-024-01717-3)
Supplement: Supplementary file 1 — Supplementary file1 (DOCX 117 KB) [file 11121_2024_1717_MOESM1_ESM.docx]

**Index of Supplemental Materials**

1. Supplementary Text
   1. Detailed Randomization and Data Collection Procedures
   2. Detailed Intervention Description
   3. Comparison Condition Details
   4. Detailed Measures
   5. Detailed Information on Implementation Monitoring and Integrity
   6. Detailed Description of Analytic Procedures
   7. Supplemental Results
   8. Supplemental References
2. Supplemental Tables
   1. Supplemental Table A. Intervention Curriculum Overview
   2. Supplemental Table B. Analytic Sample
   3. Supplemental Table C. Student Sample Demographic Characteristics Comparison
   4. Supplemental Table D. Comparison of Differential Attrition at Wave 6 by Condition
   5. Supplemental Table E. Child Outcome Scores by Time and CSP Intervention Group
   6. Supplemental Table F. School Outcomes by year and CSP Intervention Group
   7. Supplemental Table G. Bivariate Correlations at Baseline
   8. Supplemental Table H. HLM Results: SEL Skills; Social Problem Solving, Self-Efficacy, Teacher Overall Rating
   9. Supplemental Table I. HLM Results: Empathic Concern, Classroom Peer Community, Life Satisfaction
   10. Supplemental Table J. Student’s Behavior and Adjustment in School
   11. Supplemental Table K. School Level Achievement and Discipline
3. Supplemental Figures
   1. Supplemental Figure A. CONSORT Diagram
   2. Supplemental Figure B. Intervention effect on VESIP Social Information Processing composite score moderated by school poverty level: means by wave for students attending high poverty schools
   3. Supplemental Figure C. Intervention effect on VESIP Social Information Processing composite score moderated by school poverty level: means by wave for students attending low poverty schools.
   4. Supplemental Figure D. Intervention main effect on Self-efficacy means by wave.
   5. Supplemental Figure E. Intervention main effect on Sense of Peer Community: means by wave.
   6. Supplemental Figure F. Intervention effect on teacher-rated Conduct Problems moderated by school poverty level: means by wave for students in high poverty schools.
   7. Supplemental Figure G. Intervention effect on teacher-rated Conduct Problems moderated by school poverty level: means by wave for students in low poverty schools.
   8. Supplemental Figure H. Intervention effect on teacher-rated prosocial behavior moderated by school poverty level: means by wave for students in high poverty schools.
   9. Supplemental Figure I. Intervention effect on teacher-rated prosocial behavior moderated by school poverty level: means by wave for students in low poverty schools
   10. Supplemental Figure J. Intervention effect on teacher-rated prosocial behavior moderated by initial grade level: means by wave for students who entered the study in grades K-2.
   11. Supplemental Figure K. Intervention effect on teacher-rated prosocial behavior moderated by initial grade level: means by wave for students who entered the study in grades 3-5.
   12. Supplemental Figure L. Intervention effect on teacher-rated prosocial behavior moderated by student gender: means by wave for boys.
   13. Supplemental Figure M. Intervention effect on teacher-rated prosocial behavior moderated by student gender: means by wave for girls

**Detailed Randomization and Data Collection Procedures**

**Randomization.** All eligible elementary schools were stratified using cluster analysis into subgroups with comparable characteristics based on proportion of white students and of student free and reduced lunch. These two factors were used for blocking because other characteristics did not provide further significant differentiation. Participating schools were located into these strata for randomization within clusters. 25 schools were randomized in this manner in the first cohort (13 intervention, 12 comparison) and 17 in the second (9 intervention, 8 comparison). To ensure transparency and trust, the randomization lottery was conducted in person and overseen by district administration. On the day of lotteries, district administrators and school principals gathered, and each school’s name was placed in a bucket with other participating schools from a cluster. The schools were then drawn one at a time from the buckets and assigned to a group alternating between intervention and comparison conditions. Three schools were added to the second cohort after the initial lottery took place by request of the district. These three schools were randomly assigned to condition through a coin flip (2 intervention, 1 comparison), bringing the total sample to 45 schools (24 intervention, 21 comparison condition). In the second cohort, two schools (one intervention and one comparison condition) who fell under a state improvement plan post-randomization were encouraged by the state department of education representative to withdraw from the study to avoid additional burden on their teachers. This took place before student consent was completed or data collection began, resulting in a sample of 43 schools (23 intervention and 20 comparison condition). A CONSORT diagram with details on the school and student samples is provided in Figure A of this supplemental material.

**Data Collection Procedures Description**

***Child Assessments.*** Child assessments were completed in two sessions. Session A included computer-based tests (SELWeb and VESIP) that were completed on laptops with one trained research assistant per five children to provide instructions and answer questions. During Session B, students rotated through other assessments. Student self-report surveys were administered on tablets using an online survey platform (Qualtrics) in groups of five children at a table with one research assistant trained to provide instructions and answer questions. At the first day of data collection for each wave, students affirmed their assent before participating. Students who refused to participate were invited to participate again on the next visit unless they withdrew from the study. A make-up day was scheduled at each wave to assess students who were absent or unavailable at the originally scheduled time. Each year, project staff communicated with families via phone, email, and post-cards to thank them for participation, answer questions, check for school transfers, and withdraw students from the study if requested (n=38).

***Teacher Surveys.*** At the beginning of each year, during teacher in-service time or staff meetings, project staff provided an overview of the project and data collection activities and explained the request for teacher surveys about participating students. Email was used to contact teachers so they could utilize an online survey portal (Qualtrics) to complete assessments for their participating students. Research staff delivered reminder notes and small nominal incentives (e.g., post-its, a granola bar, stickers) in their mailboxes to encourage participation when the survey period began at each wave. Teachers received $5 per completed survey. Low initial participation rates in the online surveys prompted the inclusion of paper survey options in year 2 (Cohort 1 Wave 4 and Cohort 2 Wave 2). Research staff delivered paper surveys and reminders to teacher mailboxes in addition to sending online survey instructions to teacher emails.

**Detailed Intervention and Comparison Condition Description**

**Standards Consulted.** The CSP *Flourish* curriculum authors consulted with available state and national standards to guide the development and revisions of the curriculum. The CASEL Framework and 2013 CASEL Guide (CASEL, 2012) were the primary source for determining SEL skills and understandings that would be covered by the curriculum. At the time, Illinois was one of the only states to have elementary level SEL standards, so we also used these standards to inform the development of curriculum content (Illinois State Board of Education, 2004). Because the curriculum was designed to be broader than SEL and address a more comprehensive set of health and wellness content, we also consulted the National Health Education Standards (Joint Committee on National Health Education Standards, 2007) to ensure that the curriculum was supporting, at least in part, the development of the understandings and skills central to health education. During the piloting and curriculum revision process, the Kentucky Core Academic Standards for Practical Living (Health and PE; Kentucky Department of Education, 2013) were consulted to align content as much as possible, especially with the standards addressing health and social-emotional wellbeing.

**CSP *Flourish* Curriculum Overview**. Unit 1 provides an introduction framing learning within an exploration of how to build a caring and compassionate community, Units 2-6 provide the core of the curriculum, focusing on intrapersonal skills (Units 2-3) first and then interpersonal skills (Units 4-6). Unit 7 engages students in applying their learning by developing a service project to contribute to their community. Each lesson includes interactive SEL activities such as group discussions, games, role-plays, partner activities, and self-expression through journaling and art. Mindful awareness practices (“Mindful Moments”) aligned with the SEL objectives are central to each lesson. The curriculum includes a variety of Mindful Moments (designed to cultivate focused attention, self-awareness, self-regulation, and appreciation for self, others, and the world around us), which are linked with the development of children's executive functions (EFs—cognitive control abilities reliant on the prefrontal cortex (PFC) that help organize, sequence, and regulate behavior), stress regulation, well-being, and prosocial behavior (MLERN, 2012). Each lesson also integrates opportunities for physical activity, primarily (but not limited to) a range of “Mindful Movement” practices inspired by yoga and creative movement, designed to enhance body awareness, balance, proprioception, strength, agility, and embodied self-regulation. In the Kindergarten-1^st^ grade curriculum, lessons include Mindful Movement in the form of “Moving Storytime.” During Moving Storytime, teachers lead learners through a story that explores the SEL objectives of the lesson, embodying the characters and acting out the narrative through movement. Table A provides an overview of Units in the *Flourish* curriculum, example student-centered learning goals from one lesson in each unit, and examples of one Mindful Moment and Mindful Movement practice central to the unit. Please contact [csp@virginia.edu](mailto:csp@virginia.edu) to access the full scope and sequence, curriculum manuals, and other intervention materials.

**Intervention Training and Implementation Supports.** All curriculum training was provided by the curriculum developers and implementation coaches. Implementation coaches were certified teachers experienced in social-emotional learning, health/PE/wellness education, mindfulness, and/or mindful movement. CSP PL teachers received 5 days of training in the summer and a 1-day booster training mid-year during each year of the study. Teachers had the choice to receive professional development credit or pay for their attendance in the summer training. The first two days of the summer institutes and the first half of the mid-year booster consisted of evidence-based professional learning (adapted from Cultivating Awareness and Resilience in Education, Jennings et al., 2017) to support educators’ own social-emotional competence and professional wellbeing. This provided psychoeducation about emotions and stress and experiential practice with mindfulness and compassion. The 3-5th day of the summer institute and the second half of the mid-year booster were focused on curriculum content and instruction. Training was heavily experiential with participation in demonstration lessons, Mindful Moment practices, and Mindful Movement with opportunities for practice teaching and feedback.

Additional implementation supports included Professional Learning Community (PLC) meetings, coaching, consultation, and a school-based program to support staff wellbeing (CALM for Educators). PLC meetings were offered monthly for professional development credit or stipend. These meetings included SEL, Mindful Moment, and/or Mindful Movement activities facilitated by implementation coaches, discussions of curriculum content, and time for planning and problem solving with peer teachers. Coaches visited schools at least once per month, but often more frequently, to provide instructional support. Coaches observed classes, engaged in conversations related to implementation goals and integrity of implementation, provided feedback, and were available as requested for modeling or co-teaching lessons. Coaches and the Project Director met with principals annually to provide consultation related to implementation and integration into the culture of the school. Finally, the Community Approach to Learning Mindfully (CALM) for Educators program was implemented in intervention schools in the morning before school as an optional support for staff wellbeing. This was an opportunity for educators and other staff to engage in mindfulness and movement practices similar to those being implemented for students. Participation in CALM varied widely across schools. For example, in the first year, the total number of individual staff members who participated in CALM at least once ranged from 3-24 (M=11.3, SD=5.4), and the total number of sign-ins to CALM sessions across the year and across all participants in a school ranged from 7-180 (M=89.4, SD=48.2).

**Comparison Condition Details**

**Curriculum.** Schools in the comparison EAU condition continued to implement their Practical Living and SEL curricula as usual. Practical Living classes were generally 50 minutes once per week, focused primarily on physical education. Health, nutrition, and other wellness content was often integrated both in these PL classes and in classroom instruction in order to meet the state standards for PL. SEL implementation was generally integrated with core instruction, taught as guidance lessons, or as part of morning meetings. All schools, comparison and intervention, received PBIS professional development and implemented PBIS per state mandates. Based on survey responses from school administrators, comparison schools were implementing several different SEL programs during the course of the study, including Second Steps, Care for Kids, PATHS, Leader in Me, Sanford Harmony, and unspecified character education and guidance curricula. Care for Kids and Second Steps were widely implemented districtwide. During the first year of the RCT, the district began rolling out training and implementation support for PATHS and Restorative Practices. Several comparison schools indicated that teachers were implementing mindfulness practices to some extent, and one comparison school implemented the MindUp program for the duration of the study.

**Comparison School Training and Implementation Support.** Comparison school PL teachers had access to a district level PL specialist and resource teacher that provided professional development and on-site instructional coaching. PL teachers were required to participate in at least 18 hours of professional development in addition to monthly PLCs and school-based professional development. In addition to their in-school PLCs with other special area teachers, PL Teachers often participated in cross-site PLCs with PL teachers from other schools (this was the model based on which the CSP PLCs were implemented). The financial incentive provided to comparison schools annually was often applied to purchase new PE or wellness equipment. As part of the incentives to comparison schools, the CSP implementation coaches offered PD to the comparison schools. CSP coaches offered PD similar to what the CSP teachers received, but without specific content on the CSP curriculum, For example, 1 hr sessions on mindfulness in classroom management and on integrating movement into the classroom. This was rarely utilized (about 4 times across the 4 years of the study). CSP Coaches also offered these PD opportunities at a district-wide professional development conference in the summer available to all teachers in the district.

**Detailed Measures Description**

**Student SEL Skills.** Student SEL skills were assessed using direct performance measures, student self-report, and teacher ratings of students, all described below. The primary direct performance measure, SELWeb is an on-line direct assessment with activity-based modules targeting various facets of children’s SEL skills, including emotion recognition, self-control, social problem solving, and social cognition (McKown et al., 2016). The entire SELWeb measure was administered to grades K-3, but Grades 4-5 only received the emotion recognition and self-control modules. Because SELWeb was optimized for grade K-3, a related online direct assessment, VESIP (Virtual Environment for Social Information Processing; Russo‐Ponsaran et al., 2017), was used for age-appropriate assessment of social problem solving in grades 4-8. Specific measures for each SEL construct are detailed below:

***Emotion Recognition*.** Based on studies of nonverbal communication (Nowicki & Duke, 1994), the SELWeb emotion recognition module taps the capacity to comprehend the emotions of others, requiring participants to identify appropriate emotion labels for a series of 40 faces. Facial expressions of emotion are balanced across race and gender, and participants respond to the question “How is the child feeling?” by choosing from 5 responses (happy, sad, angry, scared, or just okay). Responses are assigned a score of 2 for correct labels, 1 for labeling an emotion as neutral (just okay), and 0 for an incorrect label. Across 40 trials in Wave 1 α=.86).

***Self-Management.*** SELWeb includes two modules for “self-control,” designed to tap into the cognitive processes necessary for self-restraint, emotion regulation, and delayed gratification in pursuit of an objective (Duckworth, 2011). The first module tests participants’ *Frustration Tolerance* by scoring the number of correct object-matching trials students complete despite a frustrating “glitch” in the computer program that appears to slow down the test's ability to accept responses while a timer ticks down on the screen. Participants may complete up to 23 trials in 90 seconds if they perseverate on the task, receiving 1 point for each correct response. The second SELWeb self-control module, *Choice Delay,* taps into executive function, and specifically delay of gratification, in the context of a video game where rockets are sent to the moon to earn points. Participants are given a demo that explains the slowest rocket earns the most points while the fastest rocket earns the fewest points, and told the goal is to earn the most points possible in ten trials. Because the game is untimed, participants will earn the maximum (30) points by always choosing the slowest rocket. The two self-control modules, *Frustration Tolerance* and *Choice Delay,* were administered to grades K-5, and total scores for the two modules were utilized in analyses.

In addition, we utilized the Flanker (Diamond et al., 2007) test, designed to assess attention and inhibitory control, for all grade levels. Participants are shown images of fish pointing in different directions on the screen of a tablet and must identify the direction of the target fish in a series of three challenges with varying instructions (Flanker, Reverse Flanker, and Mixed trials). Average overall response time (RT) was calculated in milliseconds (ms) for accurate, non-impulsive responses within each trial. We recorded the average RT overall and for the mixed trial (most challenging).

***Social Problem-Solving.*** Targeting the ability to logically consider social issues, the SELWeb and VESIP assessments of Social Problem Solving are both grounded in social information-processing research (Bauminger, et al., 2005; Crick & Dodge, 1994). For grades K-3, SELWeb presents vignettes with illustrated social situations and asks a series of questions assessing participants’ social reasoning (e.g. “What do you want to happen?”). Because there were multiple forms of the test, students’ scores at each wave were standardized based on the baseline average for the form that they took. These standardized scores for social goals (6 items) and solution preference (6 items), were combined in a mean score representing Social Problem-Solving. The test developers made changes to the test that prevented scoring equivalence for the final two waves of data collection for each cohort, so those waves were not included in the analyses. For grades 4-8, VESIP assesses similar processes through animated social situations (e.g., ambiguous provocation, peer entry into a group), in which an avatar represents the participant. To assess their social reasoning, participants are asked questions about how they would respond to each of the 5 scenarios. The VESIP Social Information Composite is an overall mean of the scores across the 5 scenarios for Problem Identification, Social Goals, and Solution Preference. We used a version of this composite score modified to align with our research questions.

***Social Self Efficacy.*** For Grades 4-8 Social Self Efficacy was assessed in VESIP. For each of the 5 social scenarios, participants are reminded of which solution they chose and asked to rate how confident they are that they could enact their preferred solution using a slider from “not at all sure” (scored 0) to “very sure” (scored 5). The mean score for Social Self-Efficacy was calculated across the 5 scenarios (Wave 1 α=.48).

***Teacher Rating of Student SEL Skills.*** We also measured student SEL skills for Grades K-5 using ratings of the student sample by classroom teachers (not the CSP instructors) using the Teacher Report on Students’ Social and Emotional Competence (CASEL & the American Institutes for Research, 2013; Kendziora & Osher, 2016). Teachers rate students on 20 items comprising 5 subscales (Self-Awareness, Self-Management, Social Awareness, Relationship Skills, and Responsible Decision Making) with responses on a 4-point scale ranging from rarely to almost always (Wave 1 internal consistency for the subscales ranged from α=.90 - α=.98). Prior psychometric testing in 7 districts with response rates averaging 36-64% showed good Rasch reliability (.82-87) and unidimensionality of subscales. Examination of our sample data from the first wave showed correlation among scales approaching .90 and exceeding in some cases, therefore we recorded an overall mean Teacher Rating of SEL skills per child.

***Empathy.*** We assessed self-reported empathy using the Interpersonal Reactivity Index (IRI; Davis, 1983) adapted for use with elementary-age children (Catherine & Schonert-Reichl, 2011). This self-report survey was administered to Grades 4-8 by tablet using Qualtrics. The empathic concern subscale (mean of 7 items, Wave 1 α=.79) assesses concern for others (e.g., ‘I often feel sorry for kids who don’t have the things I have’) and the perspective taking subscale (mean of 7 items; Wave 1 α=.75) assesses inclination to take another’s point of view (e.g., ‘I try to look at everybody’s side of a disagreement before I make a decision’) with scores ranging from 1 (not at all like me) to 5 (always like me).

**Student Subjective Well-being**. Two self-report survey measures targeted students’ subjective wellbeing. *The Students’ Life Satisfaction Scale* (SLSS, Huebner, 1991) is a brief unidimensional assessment of general satisfaction with life that has previously demonstrated good internal consistency, test-retest reliability, and validity. The *SLSS* was administered to Grades 3-8 using Qualtrics on a tablet. Students responded to 7 statements, such as “I have what I want in life” and “My life is going well” on a 4-point scale ranging from “really false” to “really true" (Wave 1 α=.77). *Sense of Peer Community* was assessed using a measure of sense of community in school (Battistich, et al. 1997) adapted to measure the sense of community among peers, specifically (Madill, et al., 2014). Using a Qualtrics survey administered by tablet, students in Grades 4-8 rated 5 items (on a 5-point Likert scale ranging from never to always) assessing their perceptions of supportiveness and community (e.g., “kids in my classroom help each other”) among peers in their classroom (Wave 1 α=.83).

**Student Behavior and Adjustment in School.** Students’ behavior and adjustment in school was measured by reports from Grades K-5 classroom teachers (not CSP teachers). Teachers reported on student behavioral adjustment using the Strengths and Difficulties Questionnaire (SDQ; Goodman, 1997). The SDQ teacher report has demonstrated good reliability and validity in multiple applications (Van Den Heuvel et al., 2017). Each item on the SDQ is rated on a 3-point Likert scale (0=not true, 1=somewhat true, 2=certainly true), with higher scores indicating greater difficulties or strengths. For this study, we used three subscales: *Emotional Symptoms,* which assesses symptoms of depression, anxiety, and somatic complaints (5 items, Wave 1 α=.79); *Conduct Problems, which assesses* oppositional behavior, aggression, and rule-breaking behavior (5 items, Wave 1 α=.84), and *Prosocial Behavior*, which measures positive social behaviors such as helping, sharing, and empathy (5 items, Wave 1 α=.86)*.*

*Academic Competence* was measured for students in K to grade 5 using the Academic Competence Evaluation Scale-Short Form (ACES-TS; Anthony & DiPerna, 2018). Teachers use a 5-point Likert scale (where 0=”never” and 4= “almost always”) to rate students’ skills, attitudes, and behaviors that contribute to school success on 39 items that make up subscales for motivation (e.g. “is motivated to learn”), engagement (e.g. “volunteers to answer questions”, study skills (e.g. “corrects own work”), and interpersonal skills (e.g. “listens to what others have to say”). The mean of these 4 scales is used to generate an overall score for the ACES, *Academic Enablers*, which has demonstrated reliability and validity in previous research (Anthony & DiPerna, 2018). For this study, we used the Academic Enablers overall score (Wave 1 α=.90)*.*

**Detailed Information on Implementation Monitoring and Integrity**.

The curriculum was implemented by trained teachers in a coaching model. Coaches regularly visited classrooms to observe, provide feedback, and set goals toward continuous improvement for integrity of implementation. The curriculum was implemented across the full school year in all schools. Fidelity observations were conducted 3 times per year. Fidelity observations were conducted by two implementation specialists who did not directly coach teachers. Observations included adherence to the curriculum material among other dimensions of implementation quality. In this paper, we report on adherence to the curriculum as a proportion of 9 lesson elements observed. Observers counted each element as present or absent during the class period observed. Example lesson elements include “Calming and Focusing” practice, core SEL activity, lesson-specific mindful movement, and take-home practice.

Fidelity observations were available for Cohort 1 teachers for their second year of implementation and for Cohort 2 teachers for both years of implementation. Across all fidelity observations, teachers had a mean level of adherence to curriculum that ranged from .17-.96. The average score was .73, SD=.16 (Median = .78) indicating substantial adherence to the curriculum.

**Detailed Explanation of Analytic Models**

Inferential analyses were based on 3-level longitudinal hierarchical linear models (HLM). A growth curve for each child that estimated their skill level and linear rate of change over time was calculated. Models accounted for the nesting of children in schools at the time of random assignment by estimating school-level random intercepts and slopes from the individual coefficients. Time was centered at the end of year 2 (i.e., time 4) so main effect parameter estimates describe growth centered at the end of the intervention period. The unconditional fixed effect model included cohort and the child’s intervention condition determined by whether they attended a school randomly assigned to CSP in the first year of the study with linear and quadratic change terms over time.

Next, we calculated the conditional model adding terms for 1) the proportion of the school that received a free- or reduced-price lunch (FRPL), 2) proportion white, and 3) proportion Hispanic as covariates because baseline equivalency tests indicated nonsignificant differences. We also added interaction terms for three hypothesized moderators 1) the poverty status of the school randomly assigned (categorized as high poverty defined as 80% or more of students free- or reduced-price lunch), early vs later grade of student at trial outset (K-2 v 3-5), and gender. The three moderators were crossed with intervention condition, time, and time squared. We dropped non-linear growth and non-linear moderation (time-squared) interactions from final models if not significant in initial runs. The model for this and other analyses are:

Model for estimating student effects (for the ith child at the jth time point at the kth school):

Level 1: Y_ijk_ = B_0ik_ + B_1ik_ Time_0ijk_ + B_2ik_ Time_0ijk_^2^ + e_0ik_

Level 2: B_0ik_ = b_00k_ + + e_0k_

B_1ik_ = b_10k_ + e_1k_

B_2ik_ = b_20k_

Level 3: b_00k_ =d_00_ + d_01_ CSP_k_  + b_02k_ Cohort_ik_ + z_0k;_

b_10k_ =d_10_ + d_11_ CSP_k_ + z_1k;_

b_20k_ =d_20_ + d_21_ CSP_k_

Model for estimating gender when missing, Levels 2 and 3 for this model are:

Level 2: B_0ik_ = b_00k_ + b_01k_ Initial Grade_ik_ + b_02k_ Gender_ik_ + b_03k_ Cohort_ik_ + e_0k_

B_1ik_ = b_10k_ + b_11k_ Initial Grade_ik_ + b_12k_ Gender_ik_ + e_1k_

B_2ik_ = b_20k_ + b_21k_ Initial Grade_ik_ + b_22k_ Gender_ik_

Level 3: b_00k_ =d_00_ + d_01_ CSP_k_  + d_02_ School Poverty_k_  + d_03_ CSP_k_  x School Poverty_k_  +

d_04_ School FRPL_k_ + d_05_ School White_k_ d_04_ School Hispanic_k_ z_0k;_

b_10k_ =d_10_ + d_11_ CSP_k_  + d_12_ School Poverty_k_  + d_13_ CSP_k_  x School Poverty_k_  + z_1k;_

b_20k_ =d_20_ + d_21_ CSP_k_  + d_22_ School Poverty_k_  + d_23_ CSP_k_  x School Poverty_k_

b_01k_ =d_01_ + d_01_ CSP_k_  ; b_11k_ =d_11_ + d_11_ CSP_k_ ; b_21k_ =d_21_ + d_21_ CSP_k_

b_02k_ =d_02_ + d_02_ CSP_k_  ; b_12k_ =d_12_ + d_12_ CSP_k_ ; b_22k_ =d_22_ + d_22_ CSP_k_

b_03k =_ d_03k_

Because we could not obtain gender information for all children (17% missing), we used multiple imputation across 20 imputed data sets to render that variable for models. These models were applied for all student direct assessments and self-reports. For teacher ratings, we added a clustering variable because some teachers rated multiple children in their classrooms.

For school level archive comparisons, 2-level HLM analyses were utilized. The level 1 model described change over time in the school-level measures of academic achievement and discipline referrals and the level 2 model added CSP intervention condition and cohort. Preliminary models allowed for quadratic time, but the quadratic term was dropped because it was not significant in preliminary models. Model for school level comparisons:

Level 1: Y_ijk_ = B_0ik_ + B_1ik_ Time_0ijk_ + e_0ik_

Level 2: B_0ik_ = d_00_ + d_01_ CSP_k_  + b_02k_ Cohort_ik_ + z_0k;_

B_1ik_ = d_20_ + d_21_ CSP_k_

**Comparisons for Baseline Equivalence and Differential Attrition**

**Baseline Equivalence.** Consent rates were summarized at the classroom level and analyses of variance that accounted for nesting of classrooms in schools tested whether consent rates varied by grade, demographic characteristics, and CSP group. Classroom level consent rates were lower in grade 5 (47%) than other grades (50-56%) but did not differ by condition. School level consent rates were significantly higher for schools with a larger proportion of White students (r = .41) and lower for schools with a larger proportion of Black children (r = -.47) and for schools with higher rates of children with FRPL (r = -.44), none of these differed by condition. Consent rates among schools with higher proportion Hispanic children was significantly by condition but only slightly more in the CSP (r = .25) than in the comparison group (r = -.06).

Demographic **c**haracteristics of the schools and students at recruitment were compared to see if they differed by intervention condition (Supplemental Table C). No significant differences were found, except that there were more students from high poverty schools in the comparison condition (33% compared to 24% CSP condition). Because there was a variation between conditions in proportion of schools whose students received free or reduced-price lunch and because the proportion who were Hispanic were each more than 15% of a standard deviation, these two school demographic characteristics were included in models as covariates and were included in analyses of covariates (What Works Clearinghouse, 2018).

**Differential Attrition**. To assess for differential loss, whether the student participated in the wave 6 data collection was utilized, with comparisons on all student sample demographic and outcome variables as measured at baseline. We compared at this wave because of COVID disruption of assessment for the final year of data collection (waves 7 & 8) for the second cohort. Results shown in Supplemental Table D revealed significant differences in retention by intervention condition on one demographic characteristic and two child outcomes. Proportionately more White students were retained in the EAU comparison condition (46%) than in the CSP condition (41%). Mean score on Self Efficacy at Wave 1 among students in the EAU comparison condition was significantly higher for those retained (M=4.00) than lost (M=3.84), but for the CSP condition was significantly higher for those lost than (M= 3.98) than retained (M=3.88). Mean score on Empathic Concern at Wave 1 among students in the EAU comparison condition was not significantly different for those retained (M=3.67) and those lost (M=3.70), but in the CSP condition the average score was significantly higher for those retained (M= 3.80) than those lost (M=3.58). Each of these differences are quite small in magnitude.

**Preliminary Scan of Distributions and Correlations.** Prior to model runs, descriptive inspection of distributions and data completeness were conducted. In addition, we examined correlation among indicators theorized to measure similar constructs, including modifying scaling and utilizing summary scores where subscales were highly intercorrelated. Those procedures are specified in the measurement descriptions. Means for child and school variables by wave are presented in Supplemental Tables E and F respectively. Bivariate correlations among outcome measures at baseline are provided in Supplemental Table G.

**Supplemental References**

Bauminger N., Edelsztein H. S., Morash J. (2005). Social information processing and emotional understanding in children with LD. *Journal of Learning Disabilities, 38*(1), 45-60. <https://doi.org/10.1177/00222194050380010401>

Collaborative for Academic, Social, and Emotional Learning. (2012). *2013 CASEL guide: Effective social and emotional learning programs—Preschool and elementary school edition*. Chicago, IL: Author.

Crick N. R., Dodge K. A. (1994). A review and reformulation of social information-processing mechanisms in children’s social adjustment. *Psychological Bulletin, 115*(1), 74-101. <https://doi.org/10.1037/0033-2909.115.1.74>

Duckworth A. L. (2011). The significance of self-control. *Proceedings of the National Academy of Sciences of the United States of America, 108*(7), 2639–2640. <https://doi.org/10.1073/pnas.1019725108>

Illinois State Board of Education (2004). Illinois Social and Emotional Learning Standards. Retrieved 2014 from <https://www.isbe.net/sel>.

Joint Committee on National Health Education Standards. (2007). National Health Education Standards, Second Edition: Achieving Excellence. Washington, D.C.: The American Cancer Society. Retrieved from https://www.shapeamerica.org/MemberPortal/standards/health/2007-he-standards.aspx

Kendziora, K. & Osher, D. (2016). Promoting children’s and adolescents’ social and emotional development: District adaptations of a theory of action. *Journal of Clinical Child & Adolescent Psychology, 45(6),* 797-811. <https://doi-org/10.1080/15374416.2016.1197834>

Kentucky Department of Education (2013). KYCAS Practical Living Standards (Health and Physical Education). Retrieved 2014 from <http://education.ky.gov/curriculum/docs/Documents/KCAS%20-%20June%202013.pdf>

Mind and Life Education Research Network (MLERN). (2012). Contemplative practices and mental training: Prospects for American education. *Child Development Perspectives, 6*(2), 146-153. <https://doi.org/10.1111/j.1750-8606.2012.00240.x>

Nowicki S. & Duke M. P. (1994). Individual differences in the nonverbal communication of affect: The diagnostic analysis of nonverbal accuracy scale. *Journal of Nonverbal Behavior, 18*, 9-35. <https://doi.org/10.1007/BF02169077>

van den Heuvel, M., Jansen, D. E., Stewart, R. E., Smits-Engelsman, B. C., Reijneveld, S. A., & Flapper, B. C. (2017). How reliable and valid is the teacher version of the Strengths and Difficulties Questionnaire in primary school children? *PloS one, 12(4),* e0176605. https://doi.org/10.1371/journal.pone.0176605

| Supplemental Table A. CSP *Flourish* Curriculum Unit Overview, Example Lesson Goals by Grade Level, and Example Practices | | | | |
| --- | --- | --- | --- | --- |
|  | Example Student Centered Learning Goals | | | Example Practices |
|  | Grades K-1 | Grades 2-3 | Grades 4-5 |  |
| Unit 1 Compassion (4 lessons) | I can show myself compassion by caring for my body. (Lesson 2) | I can work with a partner to demonstrate an act of compassion for others (Lesson 3) | I can identify a compassionate solution to a difficult situation. (Lesson 3) | Mindful Moments: *Calming & Focusing* (3 breaths and mindful listening with chime)  Movement: Mini-Sequences (linking breath and movement) |
| Unit 2 Self-Awareness (7 lessons) | I can tell you whether an emotion is comfortable or uncomfortable. (Lesson 3) | I can describe what makes me feel like my best self. (Lesson 5) | I can identify what triggered a feeling. (Lesson 2) | Mindful Moments: *Checking-in* (present-moment awareness with senses, emotions, etc.)  Mindful Movement: Standing poses (focusing on body awareness) |
| Unit 3 Self-Management (7 lessons) | I can demonstrate finding my Anchors to calm and focus my mind. (Lesson 1) | I can identify times when it might be challenging for me to be my best self (Lesson 6) | I can describe strategies to help me be my best self when I experience strong emotions. (Lesson 3) | Mindful Moment: *Letting Go Breath* (slow exhalation)  Mindful Movement: Balance poses (practicing physical self-regulation) |
| Unit 4 Social Awareness: Empathy and Perspective-Taking (6 lessons) | I can use curiosity to notice and learn about the spaces and people around me. (Lesson 6) | I can recognize that other people may see, think, feel, or do things differently than I do (Lesson 2) | I can explain why two people might have different perspectives on the same situation. (Lesson 2) | Mindful Moment: *Gratitude and Appreciation* (for another person)  Mindful Movement: Twists (using physical posture to explore point of view) |
|  |  |  |  |  |
| Unit 5 Relationships Skills (8 lessons) | I can help a friend feel welcomed by greeting them and introducing myself. (Lesson 1) | I can use my body, my ears, and eyes to show that I am listening. (Lesson 5) | I can name one or more strategies to de-escalate conflict with a peer. (Lesson 4) | Mindful Moment: *Kind Mind* (practicing kind thoughts and intentions for self and others)  Mindful Movement: Partner poses (practicing care and communication) |
| Unit 6 Social Awareness: Community (6 lessons) | I can name one member of my school community and tell you what their role is. (L1) | I can identify community groups to which I belong. (L2) | I can practice cooperating with a group to find a consensus solution. (L4) | Mindful Moment: *Visualizing a Flourishing Community*  Mindful Movement: Group poses (practicing collaboration) |
| Unit 7 Community Compassion Projects: In all grade levels, students collaboratively identify a need within their community and design a project as a compassionate response to this need. | | | |  |

| Supplemental Table B. Total Participants in Analytic Sample at Each Year | | | |
| --- | --- | --- | --- |
|  | Total Participating Students in Analytic Samp | Proportion with Child Assessments | Proportion with Teacher Reports |
| Year 1 (Waves 1-2) | 4762 | .99 | .46 |
| Year 2 (Waves 3-4) | 3737 | .99 | .57 |
| Year 3 (Waves 5-6) | 2430 | .99 | .32 |
| Year 4 (Waves 7-8)* | 1319 | .96 | .32 |
| *Only Cohort 1 contributed Year 4 data due to pandemic-related school closures preventing Waves 7 and 8 data collection for Cohort 2 | | | |

| Supplemental Table C. Student Sample Demographic Characteristics Comparison | | | | | | | | | | |
| --- | --- | --- | --- | --- | --- | --- | --- | --- | --- | --- |
|  | *CSP* | | | |  | *EAU* | |  |  |  |
|  | *N* | *Prop* |  | *N* | | | *Prop* |  | *Odds ratio* | *p* |
| Child Sex | 2141 |  |  | 1784 | | |  |  | 0.92 | .17 |
| Male | 1068 | 0.50 |  | 851 | | | 0.52 |  |  |  |
| Female | 1073 | 0.50 |  | 933 | | | 0.48 |  |  |  |
| Race/Ethnicity | 2079 |  |  | 1728 | | |  |  |  |  |
| White | 846 | 0.40 |  | 766 | | | 0.41 |  | 1.02 | .76 |
| Black | 755 | 0.36 |  | 582 | | | 0.34 |  | 0.89 | .09 |
| Hispanic | 246 | 0.12 |  | 245 | | | 0.14 |  | 1.23 | .03 |
| Native American/Pacific Islander | 7 | <.01 |  | 2 | | | <.01 |  | NA |  |
| Asian American | 73 | 0.04 |  | 65 | | | 0.04 |  | 1.07 | .68 |
| Multiple | 158 | 0.07 |  | 128 | | | 0.07 |  | 1.02 | .76 |
| Initial Grade |  |  |  |  | | |  |  |  |  |
| In grade K-2 in first year of study | 2144 | 0.50 |  | 2555 | | | 0.51 |  | 0.99 | .81 |
| High poverty School^a^ |  |  |  |  | | |  |  |  |  |
| School: < 80% FRPL | 2555 | 0.24 |  | 2144 | | | 0.33 |  | 0.64 | <.001 |
|  |  |  |  |  | | |  |  |  |  |
| ^a^*80% or more of students receive free/reduced price lunch*  Note: EAU=Education as Usual School; CSP Compassionate School Project School | | | | | | | | | | |

| Supplemental Table D. Comparison of Differential Attrition at Wave 6 by Condition. | | | | | | | | | | | | | | |
| --- | --- | --- | --- | --- | --- | --- | --- | --- | --- | --- | --- | --- | --- | --- |
|  |  | | | | | |  | | | | | |  | |
|  | Comparison Group | | | | | | CSP Group | | | | | |  | |
|  | Lost | | | Retained | | | Lost | | | Retained | | |  | |
|  | *N* | *Mean/Prop* | *SD* | *N* | *Mean/Prop* | *SD* | *N* | *Mean/Prop* | *SD* | *N* | *Mean/Prop* | *SD* |  |  |
| Proportion Asian American | 822 | 0.04 |  | 906 | 0.04 |  | 929 | 0.04 |  | 1150 | 0.03 |  |  |  |
| Proportion Black/African American | 822 | 0.39 |  | 906 | 0.29 |  | 929 | 0.39 |  | 1150 | 0.34 |  |  |  |
| Proportion: White | 822 | 0.36 |  | 906 | 0.46 |  | 929 | 0.39 |  | 1150 | 0.41 |  | * |  |
| Proportion: Hispanic | 822 | 0.14 |  | 906 | 0.14 |  | 929 | 0.10 |  | 1150 | 0.13 |  |  |  |
| Proportion Mixed Ethnicity: | 822 | 0.07 |  | 906 | 0.08 |  | 929 | 0.08 |  | 1150 | 0.07 |  |  |  |
| Proportion Male | 831 | 0.49 | 0.50 | 953 | 0.47 | 0.50 | 952 | 0.51 | 0.50 | 1189 | 0.49 | 0.50 |  |  |
| SW Emotion Regulation Score | 926 | 0.03 | 0.96 | 846 | 0.06 | 0.95 | 1032 | -0.07 | 1.03 | 1123 | 0.01 | 1.04 |  |  |
| SW Choice Delay Total Score | 811 | 21.45 | 4.87 | 624 | 22.20 | 4.86 | 878 | 21.26 | 5.02 | 850 | 21.92 | 5.25 |  |  |
| SW Frustration Tolerance Total | 784 | 17.15 | 2.88 | 587 | 17.53 | 2.45 | 855 | 17.23 | 2.72 | 823 | 17.34 | 2.64 |  |  |
| Flanker Overall Response Time | 535 | 859.8 | 101.6 | 577 | 853.8 | 95.90 | 593 | 854.1 | 96.20 | 658 | 862.3 | 96.62 |  |  |
| SW Social Problem Solving | 588 | 1.25 | 0.44 | 559 | 1.31 | 0.44 | 667 | 1.24 | 0.42 | 775 | 1.29 | 0.43 |  |  |
| VESIP Social Info Processing | 223 | 0.19 | 0.17 | 187 | 0.18 | 0.16 | 280 | 0.20 | 0.17 | 215 | 0.18 | 0.17 |  |  |
| SW Self Efficacy | 223 | 3.84 | 0.80 | 187 | 4.00 | 0.77 | 280 | 3.98 | 0.93 | 215 | 3.88 | 0.89 | * |  |
| Empathic Concern | 293 | 3.70 | 0.78 | 282 | 3.67 | 0.80 | 329 | 3.58 | 0.80 | 323 | 3.80 | 0.77 | * |  |
| Classroom Community | 287 | 2.38 | 0.87 | 278 | 2.45 | 0.89 | 327 | 2.37 | 0.84 | 317 | 2.42 | 0.79 |  |  |
| Conduct Problems | 287 | 0.30 | 0.42 | 56 | 0.24 | 0.41 | 422 | 0.30 | 0.45 | 124 | 0.23 | 0.39 |  |  |
| Emotional Problems | 287 | 0.27 | 0.40 | 56 | 0.26 | 0.42 | 422 | 0.35 | 0.42 | 124 | 0.33 | 0.41 |  |  |
| Prosocial Behavior | 287 | 1.55 | 0.49 | 56 | 1.43 | 0.47 | 424 | 1.56 | 0.50 | 124 | 1.55 | 0.46 |  |  |
| ACES Total Score | 284 | 2.72 | 0.91 | 55 | 2.64 | 0.77 | 420 | 2.76 | 0.85 | 124 | 2.69 | 0.89 |  |  |
| *p<.05 Group X Retained Interaction | | | | | | | | | | | | | |  |

Table E. Child Outcome Scores by Time and CSP Intervention Group

|  |  | Fall Y1^a^ | | Spring Y1 | | Fall Y2 | | Spring Y2 | | Fall Y3 | | Spring Y3 | | Fall Y4 | | Spring Y4 | |
| --- | --- | --- | --- | --- | --- | --- | --- | --- | --- | --- | --- | --- | --- | --- | --- | --- | --- |
|  |  | EAU | CSP | EAU | CSP | EAU | CSP | EAU | CSP | EAU | CSP | EAU | CSP | EAU | CSP | EAU | CSP |
| SW Emotion Recognition  (K-G3) | *N* | 1772 | 2155 | 800 | 1358 | 1408 | 1717 | 1430 | 1729 | 890 | 1203 | 729 | 857 | 484 | 455 | 155 | 191 |
|  | *Mean* | 0.04 | -0.03 | 0.22 | 0.18 | 0.23 | 0.20 | 0.35 | 0.32 | 0.52 | 0.53 | 0.48 | 0.40 | 0.96 | 0.91 | 0.96 | 0.85 |
|  | *SD* | 0.95 | 1.03 | 0.85 | 0.91 | 0.80 | 0.86 | 0.75 | 0.83 | 0.81 | 0.92 | 0.84 | 0.99 | 0.70 | 0.84 | 0.74 | 1.01 |
| SW Choice Delay Total Score (K-G4) | *N* | 1435 | 1728 | 779 | 1279 | 1351 | 1645 | 1399 | 1685 | 872 | 1176 | 716 | 839 | 482 | 455 | 155 | 191 |
|  | *Mean* | 21.78 | 21.58 | 22.11 | 21.49 | 22.06 | 21.56 | 21.29 | 21.14 | 22.20 | 21.59 | 21.07 | 20.46 | 21.87 | 22.11 | 21.36 | 20.01 |
|  | *SD* | 4.88 | 5.14 | 5.49 | 5.64 | 5.52 | 5.51 | 6.14 | 6.08 | 6.14 | 5.98 | 6.61 | 6.54 | 6.55 | 6.50 | 6.69 | 6.97 |
| SW Frustration Tolerance Total (K-G4) | *N* | 1371 | 1678 | 766 | 1246 | 1312 | 1611 | 1377 | 1661 | 866 | 1166 | 711 | 832 | 481 | 446 | 153 | 189 |
|  | *Mean* | 17.31 | 17.29 | 17.76 | 17.74 | 17.79 | 17.77 | 18.17 | 18.18 | 17.98 | 17.75 | 18.27 | 18.09 | 17.37 | 17.46 | 17.54 | 17.57 |
|  | *SD* | 2.71 | 2.68 | 2.16 | 2.29 | 2.13 | 2.09 | 1.90 | 1.93 | 1.77 | 1.89 | 1.59 | 2.11 | 1.56 | 1.33 | 1.51 | 1.36 |
| Flanker Overall Response Time (K-G8) | *N* | 1112 | 1251 | 1273 | 1586 | 1088 | 1261 | 1275 | 1528 | 746 | 1014 | 904 | 1074 | 415 | 403 | 289 | 332 |
|  | *Mean* | 856.7 | 858.4 | 835.0 | 828.9 | 819.2 | 817.1 | 789.9 | 788.8 | 790.6 | 781.9 | 763.9 | 753.8 | 774.3 | 756.6 | 737.6 | 726.1 |
|  | *SD* | 98.66 | 96.47 | 96.14 | 96.39 | 96.08 | 99.54 | 95.90 | 96.79 | 92.89 | 92.91 | 87.12 | 86.83 | 94.79 | 88.52 | 90.94 | 88.87 |
| Flankers Mixed Response Time (K-G8) | *N* | 1746 | 1968 | 1603 | 2094 | 1324 | 1562 | 1433 | 1724 | 840 | 1144 | 959 | 1149 | 448 | 433 | 313 | 349 |
|  | *Mean* | 897.6 | 901.2 | 903.3 | 900.3 | 898.8 | 893.8 | 886.4 | 882.2 | 887.9 | 872.4 | 876.5 | 862.9 | 884.4 | 856.6 | 852.8 | 841.4 |
|  | *Std* | 138.2 | 142.8 | 135.2 | 134.5 | 135.1 | 133.3 | 130.4 | 127.1 | 128.9 | 133.5 | 120.5 | 121.7 | 129.0 | 127.5 | 123.9 | 122.4 |
| SW Social Problem Solving (K-G3) | *N* | 941 | 1133 | 514 | 843 | 789 | 982 | 832 | 1020 | 315 | 352 | 318 | 351 | . | . | . | . |
|  | *Mean* | 0.02 | -0.01 | 0.11 | -0.00 | 0.19 | 0.06 | 0.21 | 0.13 | 0.27 | 0.19 | 0.35 | 0.17 | . | . | . | . |
|  | *SD* | 0.87 | 0.91 | 0.94 | 0.97 | 0.88 | 1.02 | 0.89 | 1.00 | 0.79 | 0.99 | 0.81 | 1.01 | . | . | . | . |
| VESIP Social Information Processing (G4-8) | *N* | 410 | 495 | 243 | 398 | 575 | 664 | 680 | 789 | 435 | 585 | 653 | 765 | 306 | 296 | 232 | 307 |
|  | *Mean* | 0.19 | 0.19 | 0.20 | 0.21 | 0.21 | 0.22 | 0.19 | 0.22 | 0.21 | 0.22 | 0.20 | 0.20 | 0.20 | 0.21 | 0.18 | 0.21 |
|  | *SD* | 0.16 | 0.17 | 0.20 | 0.21 | 0.20 | 0.20 | 0.20 | 0.21 | 0.21 | 0.22 | 0.21 | 0.20 | 0.20 | 0.20 | 0.19 | 0.21 |
| Social Self-Efficacy  (G4-8) | *N* | 410 | 495 | 243 | 398 | 575 | 664 | 680 | 789 | 435 | 585 | 653 | 765 | 306 | 296 | 232 | 307 |
|  | *Mean* | 3.91 | 3.94 | 4.11 | 4.09 | 4.07 | 3.95 | 4.19 | 4.16 | 4.10 | 4.00 | 4.20 | 4.20 | 4.07 | 4.11 | 4.24 | 4.35 |
|  | *SD* | 0.79 | 0.92 | 0.77 | 0.82 | 0.80 | 0.89 | 0.74 | 0.77 | 0.80 | 0.89 | 0.79 | 0.80 | 0.82 | 0.76 | 0.76 | 0.68 |
| Empathic Concern  (G4-8) | *N* | 575 | 652 | 513 | 693 | 561 | 641 | 690 | 824 | 427 | 576 | 646 | 785 | 316 | 283 | 239 | 300 |
|  | *Mean* | 3.68 | 3.69 | 3.75 | 3.64 | 3.67 | 3.63 | 3.66 | 3.65 | 3.61 | 3.63 | 3.68 | 3.72 | 3.65 | 3.57 | 3.72 | 3.63 |
|  | *SD* | 0.79 | 0.79 | 0.82 | 0.88 | 0.86 | 0.85 | 0.87 | 0.85 | 0.88 | 0.86 | 0.84 | 0.85 | 0.88 | 0.91 | 0.86 | 0.88 |
| CASEL total score  (K-G5) | *N* | 338 | 541 | 886 | 1030 | 692 | 735 | 721 | 930 | 426 | 586 | 383 | 411 | 217 | 224 | . | . |
|  | *Mean* | 2.96 | 3.04 | 3.01 | 3.01 | 3.03 | 3.03 | 3.06 | 3.01 | 3.02 | 2.98 | 3.02 | 2.97 | 3.06 | 2.98 | . | . |
|  | *SD* | 0.81 | 0.79 | 0.81 | 0.81 | 0.80 | 0.79 | 0.84 | 0.81 | 0.82 | 0.81 | 0.79 | 0.82 | 0.80 | 0.87 | . | . |
| Life satisfaction  (G3-8) | *N* | 742 | 881 | 774 | 1032 | 790 | 959 | 946 | 1154 | 639 | 853 | 777 | 900 | 454 | 433 | 136 | 185 |
|  | *Mean* | 2.15 | 2.11 | 2.13 | 2.12 | 2.17 | 2.13 | 2.14 | 2.15 | 2.17 | 2.14 | 2.14 | 2.14 | 2.09 | 2.07 | 2.09 | 2.10 |
|  | *SD* | 0.60 | 0.64 | 0.62 | 0.65 | 0.62 | 0.64 | 0.64 | 0.66 | 0.59 | 0.62 | 0.67 | 0.66 | 0.66 | 0.67 | 0.64 | 0.67 |
| Sense of Peer Community  (G4-8) | *N* | 565 | 644 | 512 | 690 | 555 | 639 | 688 | 821 | 427 | 576 | 645 | 784 | 315 | 283 | 239 | 297 |
|  | *Mean* | 2.41 | 2.39 | 2.29 | 2.27 | 2.35 | 2.31 | 2.19 | 2.21 | 2.27 | 2.29 | 2.13 | 2.23 | 2.36 | 2.33 | 2.14 | 2.16 |
|  | *SD* | 0.88 | 0.81 | 0.93 | 0.89 | 0.85 | 0.85 | 0.90 | 0.87 | 0.87 | 0.88 | 0.89 | 0.89 | 0.85 | 0.91 | 0.82 | 0.90 |
| SDQ Conduct Problems  (K-G5) | *N* | 343 | 546 | 893 | 1035 | 696 | 738 | 724 | 939 | 430 | 586 | 384 | 411 | 218 | 225 | . | . |
|  | *Mean* | 0.29 | 0.28 | 0.31 | 0.33 | 0.27 | 0.29 | 0.31 | 0.32 | 0.29 | 0.28 | 0.26 | 0.32 | 0.25 | 0.30 | . | . |
|  | *SD* | 0.42 | 0.43 | 0.47 | 0.47 | 0.42 | 0.45 | 0.45 | 0.46 | 0.47 | 0.42 | 0.41 | 0.45 | 0.41 | 0.48 | . | . |
| SDQ Emotion Problems  (K-G5) | *N* | 343 | 546 | 893 | 1035 | 695 | 738 | 724 | 940 | 429 | 586 | 384 | 407 | 218 | 225 | . | . |
|  | *Mean* | 0.27 | 0.35 | 0.32 | 0.35 | 0.31 | 0.31 | 0.34 | 0.35 | 0.32 | 0.31 | 0.34 | 0.38 | 0.32 | 0.35 | . | . |
|  | *SD* | 0.41 | 0.42 | 0.43 | 0.44 | 0.42 | 0.42 | 0.44 | 0.44 | 0.42 | 0.43 | 0.43 | 0.49 | 0.41 | 0.45 | . | . |
| SDQ Prosocial Behaviors  (K-G5) | *N* | 343 | 548 | 893 | 1035 | 696 | 739 | 724 | 940 | 430 | 586 | 384 | 411 | 218 | 225 | . | . |
|  | *Mean* | 1.53 | 1.56 | 1.57 | 1.55 | 1.57 | 1.53 | 1.57 | 1.57 | 1.53 | 1.54 | 1.58 | 1.55 | 1.58 | 1.53 | . | . |
|  | *SD* | 0.49 | 0.49 | 0.48 | 0.49 | 0.46 | 0.49 | 0.49 | 0.48 | 0.48 | 0.47 | 0.48 | 0.49 | 0.46 | 0.49 | . | . |
| Academic Engagement (ACES K-G5) | *N* | 339 | 544 | 881 | 1028 | 691 | 736 | 723 | 930 | 427 | 585 | 382 | 410 | 217 | 224 | . | . |
|  | *Mean* | 2.71 | 2.74 | 2.83 | 2.82 | 2.84 | 2.81 | 2.84 | 2.83 | 2.85 | 2.78 | 2.87 | 2.79 | 2.81 | 2.72 | . | . |
|  | *SD* | 0.89 | 0.86 | 0.84 | 0.86 | 0.83 | 0.88 | 0.89 | 0.88 | 0.87 | 0.85 | 0.82 | 0.85 | 0.83 | 0.89 | . | . |

^a^Fall Year 1 was baseline for each cohort, intervention period ended at Wave 4 (2 Years) and follow-up was for 2 year (waves 5-8). Missing cells are due to COVID preventing data collection.

|  | |  |  |  |  |  |  |  |  |  |  |  |  |  |  |  |
| --- | --- | --- | --- | --- | --- | --- | --- | --- | --- | --- | --- | --- | --- | --- | --- | --- |
| Supplemental Table F. School Outcomes by year and CSP Intervention Group | | | | | | | | | | | | | | |  |  |
|  | |  | | Spring Year 0 | | | | Spring Year 1 | | Spring Year 2 | | Spring Year 3 | | |  |  |
|  | |  | | | EAU | | CSP | EAU | CSP | EAU | CSP | EAU | CSP | |  |  |
| *Reading Mechanics -percent proficient* | | | *N* | | 20 | 23 | | 20 | 23 | 20 | 23 | 12 | | 13 | |  |
|  |  |  | *Mean* | | 43.74 | 42.87 | | 41.58 | 40.87 | 40.85 | 40.04 | 41.35 | | 38.34 | |  |
|  |  |  | *SD* | | 15.12 | 12.63 | | 14.06 | 12.98 | 15.35 | 12.33 | 11.80 | | 14.50 | |  |
| *Math Mechanics -percent proficient* | | | *N* | | 20 | 23 | | 20 | 23 | 20 | 23 | 12 | | 13 | |  |
|  |  |  | *Mean* | | 42.45 | 42.41 | | 38.33 | 37.96 | 35.63 | 32.49 | 36.56 | | 29.99 | |  |
|  |  |  | *SD* | | 15.50 | 12.90 | | 15.42 | 12.08 | 15.93 | 11.28 | 13.91 | | 11.23 | |  |
| *Percent Suspended* | | | *N* | | 20 | 23 | | 20 | 23 | 20 | 23 | 12 | | 13 | |  |
|  |  |  | *Mean* | | 6.65 | 5.70 | | 5.80 | 10.78 | 5.60 | 8.00 | 4.25 | | 4.46 | |  |
|  |  |  | *SD* | | 9.68 | 7.25 | | 6.15 | 11.98 | 5.99 | 9.02 | 4.58 | | 2.47 | |  |
| Note: School year 0 is year before school enrolled in project. EAU=Education as Usual school; CSP = Compassionate School Project school | | | | | | | | | | | | | | | | |

Supplemental Table G. Bivariate Correlations at Baseline

|  | *SW Emotion Recog.* | *SW*  *Choice Delay* | *SW*  *Frust. Tolerance* | *Flanker Overall Response Time* | *Flanker Mixed Response Time* | *SW Social Problem Solving* | *VESIP Social Information Processing* | | *VESIP Self Efficacy* | *Empathic Concern* | *Classroom Peer Community* | *SDQ Conduct Problem* | *SDQ Emotion Problems* | *SDQ Prosocial Skills* | *ACES Academic Enablers* |
| --- | --- | --- | --- | --- | --- | --- | --- | --- | --- | --- | --- | --- | --- | --- | --- |
| *CASEL Total* | 0.16*** | 0,07 | 0.13*** | 0.08 | 0.16*** | 0.22*** | -0.15 | | 0.03 | 0.25*** | 0.20 | -0.76*** | -0.32*** | 0.81*** | 0.83*** |
| *Emotion Recognition* | 1.00 | 0.19*** | 0.35*** | 0.03 | 0.25*** | 0.32*** | -0.19*** | | 0.10** | 0.07* | 0.02 | -0.11*** | -0.10*** | 0.15*** | 0.22*** |
| *Choice Delay* |  | 1.00 | 0.25*** | -0.05 | 0.14*** | 0.20*** | -0.09** | | 0.03 | 0.12*** | 0.08* | -0.02 | -0.10** | 0.02 | 0.13*** |
| *Frustration Tolerance* |  |  | 1.00 | -0.10*** | 0.18** | 0.21*** | -0.13*** | | -0.05 | 0.06 | 0.02 | -0.05 | -0.06 | 0.11** | 0.23*** |
| *Flanker Overall Response Time* |  |  |  | 1.00 | 0.73*** | 0.07* | -0.15*** | | 0.02 | 0.10** | 0.07* | -0.11* | 0.02 | 0.10* | 0.07 |
| *Flanker Mixed Response Time* |  |  |  |  | 1.00 | 0.20*** | -0.18*** | | 0.05 | 0.16*** | 0.13*** | -0.12*** | -0.08* | 0.12*** | 0.19*** |
| *Problem Solving* |  |  |  |  |  | 1.00 |  | |  | 0.50** | 0.32* | -0.16* | -0.07 | 0.19*** | 0.22*** |
| *Social Information Processing^a^* |  |  |  |  |  |  | 1.00 | | -0.07* | -0.10** | -0.11** | 0.20** | -0.08 | -0.15* | -0.09 |
| *Self Efficacy* |  |  |  |  |  |  |  | | 1.00 | 0.13*** | 0.05 | 0.01 | 0.01 | 0.09 | 0.12 |
| *Empathic concern* |  |  |  |  |  |  |  | |  | 1.00 | 0.28*** | -0.20** | -0.16* | 0.19** | 0.30*** |
| *Perceived Support* |  |  |  |  |  |  |  | |  |  | 1.00 | -0.20** | -0.09 | 0.17* | 0.17* |
| *Conduct problems* |  |  |  |  |  |  |  |  | |  |  | 1.00 | 0.26*** | -0.63*** | -0.56*** |
| *Emotion problems* |  |  |  |  |  |  |  |  | |  |  |  | 1.00 | -0.24*** | -0.36*** |
| *Prosocial skills* |  |  |  |  |  |  |  |  | |  |  |  |  | 1.00 | 0.69*** |
| *Engagement (ACES)* |  |  |  |  |  |  |  |  | |  |  |  |  |  | 1.00 |

Note * p<.05; ** p<.01; *** p<.001

^a^Social Information Processing and Self-Efficacy measured in G4-5 but Problem Solving and Social Information Processing measured in K-G3

| Supplemental Table H. HLM Results: SEL Skills; Social Problem Solving, Self-Efficacy, Teacher Overall Rating | | | | | | | | | | | | | | | | | | | | | | |
| --- | --- | --- | --- | --- | --- | --- | --- | --- | --- | --- | --- | --- | --- | --- | --- | --- | --- | --- | --- | --- | --- | --- |
|  | SW Social Problem Solving | | | | VESIP Social Information Processing | | | | | VESIP Self Efficacy | | | | | |  | | CASEL Teacher Rating | | | | |
|  | B(se) | | B(se) | | B(se) | | | B (se) | | B (se) | | | B (se) | | | B (se) | | B (se) | |  |  |  |
| Intercept | 0.228*** (0.049) | | 1.641*** (0.026) | | 0.196*** (0.009) | | | 0.196*** (0.005) | | 4.106*** (0.021) | | 4.111*** (0.022) | |  | | 2.998***  (0.038) | | 2.966*** (0.027) | | | |  |
| Cohort | 0.009 (0.059) | | 0.053*** (0.015) | | 0.018 (0.012) | | | 0.011 (0.007) | | 0.139*** (0.026) | | 0.137*** (0.027) | |  | | -0.094  (0.052) | | -0.035 (0.036) | | | |  |
| Time | 0.131*** (0.03) | | 0.198*** (0.025) | | 0.005 (0.005) | | | 0.009* (0.004) | | 0.152*** (0.02) | | 0.137*** (0.019) | |  | | -0.005  (0.012) | | -0.005 (0.012) | | | |  |
| Time^2^ | -0.051** (0.016) | | -0.068*** (0.004) | | -0.001 (0.002) | | |  | | -0.05*** (0.015) | | -0.048*** (0.014) | |  | |  | |  | | | |  |
| CSP | -0.132* (0.066) | | 0.008 (0.033) | | 0.02 (0.012) | | | 0.013 (0.007) | | -0.088** (0.029) | | -0.091** (0.03) | |  | | -0.011  (0.052) | | 0.048 (0.037) | | | |  |
| CSP * Time | -0.058 (0.039) | | 0.002 (0.031) | | 0.005 (0.007) | | | 0.006 (0.005) | | 0.003 (0.026) | | 0.002 (0.024) | |  | | -0.019  (0.015) | | -0.023 (0.016) | | | |  |
| CSP * Time^2^ |  | |  | |  | | |  | | 0.058** (0.02) | | 0.061** (0.019) | |  | |  | |  | | | |  |
| Grade |  | | -0.262*** (0.067) | |  | | | 0.027*** (0.006) | |  | | -0.059* (0.027) | |  | |  | | 0.054 (0.039) | | | |  |
| Grade * Time |  | | 0.096 (0.062) | |  | | |  | |  | |  | |  | |  | | 0.002 (0.016) | | | |  |
| CSP*Grade |  | | -0.039 (0.076) | |  | | |  | |  | |  | |  | |  | | -0.032 (0.053) | | | |  |
| CSP*Grade*Time |  | | -0.016 (0.128) | |  | | |  | |  | |  | |  | |  | | 0.008 (0.021) | | | |  |
| High Pov School |  | | 0.001 (0.031) | |  | | | -0.012 (0.014) | |  | | | -0.037 (0.052) | | |  | | 0.014 (0.069) | |  |  |  |
| Pov School * Time |  | | -0.015 (0.026) | |  | | | 0.002 (0.01) | |  | | | -0.017 (0.038) | | |  | | -0.035 (0.026) | |  |  |  |
| Pov School * Time^2^ |  | | -0.046 (0.037) | |  | | |  | |  | | |  | | |  | |  | |  |  |  |
| CSP * Pov School |  | | 0.026 (0.034) | |  | | | 0.041* (0.017) | |  | | | 0.039 (0.061) | | |  | | 0.015 (0.08) | |  |  |  |
| CSP*PovSch*Time |  | | 0.007 (0.066) | |  | | | 0.006 (0.013) | |  | | | 0.01 (0.049) | | |  | | 0.062 (0.034) | |  |  |  |
| Gender (Male=1) |  | | -0.059** (0.019) | |  | | | 0.01 (0.012) | |  | | | 0.083* (0.04) | | |  | | -0.335*** (0.038) | |  |  |  |
| Gender *Time |  | | -0.032* (0.013) | |  | | | -0.005 (0.009) | |  | | | -0.006 (0.031) | | |  | | 0.001 (0.014) | |  |  |  |
| CSP*gender |  | | 0.021 (0.018) | |  | | | 0.022 (0.015) | |  | | | -0.056 (0.054) | | |  | |  | | | |  |
| CSP*gender*Time |  | | 0.021 (0.018) | |  | | | 0.003 (0.012) | |  | | | -0.007 (0.044) | | |  | |  | | | |  |
| School % FRPL |  | -0.002** (0.001) | | 0.001** (.001) | |  |  | | 0.000 (0.001) | |  | | | | 0.001** (.001) | | -0.007*** (0.002) | |  |  |  |  |
| School % White |  | | 0.003*** (0.001) | |  | | | -0.001* (0.001) | |  | | | 0.002* (0.001) | | |  | |  | | | |  |
| School % Hispanic |  | | -0.002 (0.001) | |  | | | -0.001 (0.001) | |  | | | -0.002 (0.002) | | |  | |  | | | |  |

Note * p<.05; **p < .01; *** p<.001

Model 1 included cohort, CSP, time, and time-squared and crossed CSP with time and time-squared. Nonsignificant interactions involving time-squared were dropped

Model 2 added gender, initial grade, and high poverty schools and interacted them with CSP, time, and time-squared. Nonsignificant interactions involving time-squared were dropped

| Supplemental Table I. HLM Results: Empathic Concern, Classroom Peer Community, Life Satisfaction | | | | | | | | | | | |
| --- | --- | --- | --- | --- | --- | --- | --- | --- | --- | --- | --- |
|  | Empathic Concern | | Classroom Peer Community | | | | | Life Satisfaction | | | |
|  | B (se) | B (se) | B (se) | | | | B (se) | B (se) | | B (se) | |
| Intercept | 3.661*** (0.037) | 3.648*** (0.02) | 2.232*** (0.056) | | | | 2.224*** (0.024) | 2.188*** (0.017) | | 2.224*** (0.024) | |
| Cohort | -0.037 (0.05) | 0.000 (0.029) | -0.006 (0.075) | | | | 0.065* (0.031) | -0.015 (0.023) | | 0.065* (0.031) | |
| Time | -0.02 (0.019) | 0.001 (0.015) | -0.097*** (0.026) | | | | -0.053* (0.027) | 0.002 (0.014) | | 0.05*** (0.015) | |
| Time^2^ |  |  | 0.05*** (0.015) | | | | 0.05*** (0.015) | -0.033*** (0.007) | | -0.053* (0.027) | |
| CSP | -0.033 (0.049) | -0.008 (0.027) | 0.013 (0.076) | | | | 0.041 (0.033) | -0.018 (0.021) | | 0.041 (0.033) | |
| CSP * Time | 0.019 (0.025) | 0.016 (0.018) | 0.037 (0.035) | | | | 0.032 (0.035) | 0.01 (0.019) | | 0.032 (0.035) | |
| CSP * Time^2^ |  |  | -0.04* (0.02) | | | | -0.041* (0.02) |  | |  | |
| Grade |  | 0.073** (0.027) |  | | | | 0.184*** (0.028) |  | | 0.184*** (0.028) | |
| High Pov School |  | -0.184*** (0.054) |  | | | | -0.076 (0.061) |  | | -0.076 (0.061) | |
| Pov School * Time |  | 0.039 (0.032) |  | | | | 0.069 (0.06) |  | | 0.069 (0.06) | |
| Pov School * Time^2^ |  |  |  | | | | 0.006 (0.022) |  | |  | |
| CSP * Pov School |  | 0.086 (0.064) |  | | | | 0.016 (0.069) |  | | 0.016 (0.069) | |
| CSP*PovSch*Time |  | -0.035 (0.042) |  | | | | -0.045 (0.077) |  | | -0.045 (0.077) | |
| Gender (Male=1) |  | -0.197*** (0.04) |  | | | | 0.03 (0.042) |  | | 0.03 (0.042) | |
| Gender *Time |  | -0.04 (0.03) |  | | | | -0.029 (0.032) |  | | -0.029 (0.032) | |
| Gender *Time^2^ |  |  |  | | | | -0.007 (0.019) |  | |  | |
| CSP*gender |  | -0.064 (0.061) |  | | | | -0.038 (0.054) |  | | -0.038 (0.054) | |
| CSP*gender*Time |  | 0.062 (0.038) |  | | | | 0.046 (0.041) |  | | 0.046 (0.041) | |
| School % FRPL |  | -0.002 (0.001) | |  | | -0.002 (0.001) | | | -0.002 (0.001) | -0.002 (0.001) |  |
| School % White |  | 0.001 (0.001) |  | | 0.011*** (0.001) | | | 0.011*** (0.001) | | 0.011*** (0.001) | |
| School % Hispanic |  | 0.001 (0.002) |  | | 0.01*** (0.002) | | | 0.01*** (0.002) | | 0.01*** (0.002) | |
| Note * p<.05; **p < .01; *** p<.001  Model 1 included cohort, CSP, time, and time-squared and crossed CSP with time and time-squared. Nonsignificant interactions involving time-squared were dropped  Model 2 added gender, initial grade, and high poverty schools and interacted them with CSP, time, and time-squared. Nonsignificant interactions involving time-squared were dropped. We did not test initial grade by time interactions due to measures not being administered K-2. | | | | | | | | | | | |

| Table J. HLM Results: Student’s Behavior and Adjustment in School | | | | | | | | | |  |
| --- | --- | --- | --- | --- | --- | --- | --- | --- | --- | --- |
|  | *Conduct Problems*  *B(se) B(se)* | | *Emotion Problems*  *B(se). B(se)* | | *Prosocial Behaviors*  *B(se)* | | | *ACES Academic Enablers*  *B(se)* | |  |
| Intercept | 0.308*** (0.023) | 0.314*** (0.017) | 0.32*** (0.019) | 0.335*** (0.018) | 1.546*** (0.019) | 1.535*** (0.016) | 2.816*** (0.038) | | 2.781*** (0.029) | |
| Cohort | 0.013 (0.032) | -0.016 (0.023) | -0.008 (0.027) | -0.02 (0.024) | -0.047 0.024) | -0.024 (0.021) | -0.100* (0.051) | | -0.041 (0.039) | |
| Time | -0.004 (0.011) | -0.003 (0.011) | 0.003 (0.011) | 0.003 (0.006) | -0.007 (0.011) | -0.013 (0.012) | 0.002 (0.023) | | 0.002 (0.012) | |
| Time^2^ | -0.014** (0.005) | -0.014* (0.006) | -0.004 (0.006) |  | 0.008 (0.01) | -0.002 (0.011) | -0.013 (0.01) | |  | |
| CSP | 0.018 (0.031) | -0.002 (0.024) | 0.028 (0.026) | -0.007 (0.025) | 0.001 (0.025) | 0.003 (0.016) | -0.022 (0.051) | | 0.03 (0.039) | |
| Time*CSP | 0.006 (0.015) | 0.005 (0.015) | 0.014 (0.015) | 0.004 (0.008) | -0.008 (0.015) | 0.003 (0.014) | -0.04 (0.031) | | -0.02 (0.016) | |
| Time^2^ * CSP |  |  |  |  | -0.011 (0.013) | 0.005 (0.025) |  | |  | |
| Grade (K-2=1) |  | -0.042 (0.022) |  | -0.001 (0.021) |  | 0.045 (0.025) |  | | 0.022 (0.042) | |
| Grade * Time |  | -0.002 (0.018) |  | -0.007 (0.01) |  | 0.03 (0.023) |  | | 0.011 (0.017) | |
| Grade * Time^2^ |  |  |  |  |  | 0.03 (0.034) |  | |  | |
| Grade * CSP |  | 0.020 (0.023) |  | 0.027 (0.028) |  | -0.063 (0.033) |  | | -0.01 (0.057) | |
| Grade *CSP*Time |  | 0.012 (0.012) |  | 0.016 (0.013) |  | -0.074* (0.031) |  | | -0.009 (0.022) | |
| Grade * CSP*Time^2^ |  |  |  |  |  | -0.053* (0.023) |  | |  | |
| High Poverty School |  | 0.046 (0.045) |  | 0.025 (0.046) |  | -0.016 (0.04) |  | | -0.044 (0.074) | |
| Pov School * Time |  | 0.038 (0.024) |  | 0.009 (0.012) |  | -0.053* (0.023) |  | | -0.034 (0.025) | |
| Pov School * Time^2^ |  | -0.022 (0.053) |  |  |  | -0.001 (0.014) |  | |  | |
| Pov School * CSP |  | -0.085** (0.031) |  | -0.026 (0.054) |  | 0.018 (0.045) |  | | 0.108 (0.086) | |
| Pov School *CSP*Time |  | -0.043** (0.015) |  | -0.018 (0.016) |  | 0.084** (0.031) |  | | 0.046 (0.034) | |
| Gender (Male=1) |  | 0.111*** (0.022) |  | 0.021 (0.019) |  | -0.228*** (0.023) |  | | -0.327*** (0.041) | |
| Gender * Time |  | -0.019 (0.015) |  | 0.009 (0.015) |  | 0 (0.019) |  | |  | |
| Gender *CSP |  | 0.043 (0.03) |  | 0.003 (0.027) |  | 0.03* (0.012) |  | | -0.014 (0.056) | |
| Gender*Time *CSP |  | 0.023 (0.02) |  | -0.009 (0.012) |  | -0.008 (0.029) |  | | -0.023 (0.02) | |
| School Prop FRPL |  | 0.001 (0.001) |  | 0.004** (0.001) |  | -0.002* (0.001) |  | | -0.006*** (0.002) | |
| School Prop White |  | -0.003** (0.001) |  | 0.001 (0.001) |  | 0.001 (0.001) |  | | 0.003 (0.002) | |
| School Prop Hispanic |  | -0.003* (0.002) |  | -0.002 (0.002) |  | 0.002 (0.001) |  | | 0.005 (0.003) | |

Note * p<.05; **p < .01; *** p<.001

Model 1 included cohort, CSP, time, and time-squared and crossed CSP with time and time-squared. Nonsignificant interactions involving time-squared were dropped

Model 2 added gender, initial grade, and high poverty schools and interacted them with CSP, time, and time-squared. Nonsignificant interactions involving time-squared were dropped

| Supplemental Table K. HLM Results: School Level Achievement and Discipline | | | | | | |
| --- | --- | --- | --- | --- | --- | --- |
|  | *Reading Proficiency* | | *Math Proficiency* | | *Suspensions* | |
| Intercept | 40.206*** (1.508) |  | 35.584*** (1.761) |  | 5.852*** (1.061) |  |
| Cohort | -0.48 (2.151) |  | -3.695 (2.41) |  | -0.266 (1.575) |  |
| Time | 0.001 (0.719) |  | -2.19* (0.955) |  | -1.83 (1.093) |  |
| CSP | 0.268 (2.079) |  | -1.58 (2.429) |  | 1.501 (1.465) |  |
| Time*CSP | -0.965 (0.975) |  | -1.909 (1.299) |  | 0.667 (1.389) |  |
| School Prop FRPL | -0.279*** (0.074) |  | -0.144 (0.084) |  | 0.061 (0.066) |  |
| School Prop White | 0.435*** (0.072) |  | 0.48*** (0.082) |  | -0.199*** (0.055) |  |
| School Prop Hispanic | 0.087 (0.123) |  | 0.094 (0.142) |  | -0.281** (0.087) |  |
| Note * p<.05; **p < .01; *** p<.001 | | | | | | |
